# COP9 Signalosome Interaction with UspA/Usp15 Deubiquitinase Controls VeA-Mediated Fungal Multicellular Development

**DOI:** 10.3390/biom9060238

**Published:** 2019-06-18

**Authors:** Cindy Meister, Karl G. Thieme, Sabine Thieme, Anna M. Köhler, Kerstin Schmitt, Oliver Valerius, Gerhard H. Braus

**Affiliations:** Department of Molecular Microbiology and Genetics and Goettingen Center for Molecular Biosciences (GZMB), University of Goettingen, 37077 Goettingen, Germany; cmeiste@gwdg.de (C.M.); kthieme@gwdg.de (K.G.T.); sreen@gwdg.de (S.T.); akoehle3@gwdg.de (A.M.K.); kschmit1@gwdg.de (K.S.); ovaleri@gwdg.de (O.V.)

**Keywords:** deubiquitination, ubiquitin-specific protease, Usp15/UspA, ubiquitin, Fbx23, COP9 signalosome (CSN), *Aspergillus nidulans*, fungal development, secondary metabolism, velvet domain proteins

## Abstract

COP9 signalosome (CSN) and Den1/A deneddylases physically interact and promote multicellular development in fungi. CSN recognizes Skp1/cullin-1/Fbx E3 cullin-RING ligases (CRLs) without substrate and removes their posttranslational Nedd8 modification from the cullin scaffold. This results in CRL complex disassembly and allows Skp1 adaptor/Fbx receptor exchange for altered substrate specificity. We characterized the novel ubiquitin-specific protease UspA of the mold *Aspergillus* *nidulans*, which corresponds to CSN-associated human Usp15 and interacts with six CSN subunits. UspA reduces amounts of ubiquitinated proteins during fungal development, and the *uspA* gene expression is repressed by an intact CSN. UspA is localized in proximity to nuclei and recruits proteins related to nuclear transport and transcriptional processing, suggesting functions in nuclear entry control. UspA accelerates the formation of asexual conidiospores, sexual development, and supports the repression of secondary metabolite clusters as the *derivative of benzaldehyde* (*dba*) genes. UspA reduces protein levels of the fungal NF-kappa B-like velvet domain protein VeA, which coordinates differentiation and secondary metabolism. VeA stability depends on the Fbx23 receptor, which is required for light controlled development. Our data suggest that the interplay between CSN deneddylase, UspA deubiquitinase, and SCF-Fbx23 ensures accurate levels of VeA to support fungal development and an appropriate secondary metabolism.

## 1. Introduction

Mechanisms to defend against predators, pathogens, or harsh environmental conditions are well conserved among eukaryotes to increase chances of survival. A fast adaptive system inside cells is necessary to react to corresponding environmental stimuli. The timely coordinated translation and degradation of proteins is required to provide the correct tools for this adaptation. The ubiquitin-proteasome system (UPS) is the major pathway involved in proteome homeostasis in fungi and mammals that are evolutionarily separated already more than one billion years ago [1].

The covalent attachment and removal of posttranslational modifications alter properties of target proteins and enable a quick response to environmental stimuli without changing the whole transcriptional machinery in the cell. This can influence the activity of proteins, their binding to interaction partners, their subcellular localization, or their stability [2,3,4]. Ubiquitin is transferred to a lysine residue of the target protein by the concerted action of E1 ubiquitin-activating enzyme, E2 ubiquitin-conjugating enzyme, and the E3 ubiquitin ligase [5]. In eukaryotes, only a handful of E1 enzymes exist, whereas a few hundred E3-ubiquitin ligases account for the high number of different target proteins [6,7]. The cullin-RING ligases (CRLs) comprise the largest family of E3 ligases, and among them, the best-studied examples are the SCF (Skp1/cullin-1/Fbx) ligases [8,9]. Ubiquitin can form different homo- or heterotypic ubiquitin chains, whose functions and effects on proteins are complex [10,11]. The modification with polyubiquitin chains linked through K48 mainly targets proteins for degradation by the 26S proteasome [11,12]. The repertoire of deubiquitinating enzymes (DUBs), which reverse the ubiquitination reaction, comprises around 80 described DUBs in humans, which are divided into seven different families [13,14,15]. *Drosophila melanogaster* carries 41 and yeast 22 different deubiquitinating enzymes [6,16]. CreB is to our knowledge the only deubiquitinating enzyme studied in the ascomycete *A. nidulans.* This DUB belongs to the ubiquitin-specific protease family and is involved in carbon catabolite repression [17,18].

Human deubiquitinase Usp15 forms a small subfamily with its close relatives Usp11 and Usp4, which all share a common domain architecture. Usp15 from human erythrocytes and the ortholog Usp12 of *Schizosaccharomyces pombe* were co-purified with the COP9 signalosome (CSN) [19,20]. The COP9 signalosome is conserved between plants, fungi, and humans. In most organisms, CSN is an eight-subunit complex, but there are some variations in fungi. For example *S. pombe* contains a six-subunit CSN complex lacking the orthologs of human Csn6 and Csn8, whereas *Saccharomyces cerevisiae* has an alternative subunit composition with only five subunits. The filamentous fungus *Neurospora crassa* contains a seven-subunit CSN lacking Csn8, whereas *A. nidulans* comprises eight CSN subunits similar to human CSN. Six of the conventional eight subunits contain a proteasome, COP9, eukaryotic initiation factor 3 (PCI) domain and form a ring-like structure. The remaining two subunits contain a Mpr1, Pad1 N-terminal (MPN) domain. The C-terminal α-helices of all subunits form a helical bundle. Subunits are named Csn1/A to Csn8/H according to decreasing molecular weight [21,22,23,24].

The COP9 signalosome removes the ubiquitin-like protein Nedd8 from cullin proteins and thereby changes the activity of the ubiquitination machinery [25]. The deneddylation activity is conferred by the catalytically active MPN subunit CsnE/5, which is incorporated as the last subunit into the pre-assembled seven-subunit pre-CSN [22,26]. Deneddylation induces a conformational change of the scaffolding protein and leads to CRL complex disassembly and short-term inability to modify proteins with ubiquitin [27]. Thereby, the adaptor/receptor exchange factor CandA/1 binds to unneddylated cullins, which consists of three proteins in *A. nidulans* [28]. The deletion of single CSN subunit encoding genes leads to embryonal lethality in higher eukaryotes [29,30,31]. Single *csn* deletions of *A. nidulans* are viable and can grow vegetatively, but are impaired in multicellular development with a pleiotropic phenotype characterized by a block in sexual fruiting body formation and an altered secondary metabolism indicated by a red colony color. Several compounds, including orsellinic acid and its derivatives, were identified in *csn* mutant strains [26,32,33,34]. CSN interacts with the second deneddylase A/1 (DenA/1) in fungi and humans, which can deneddylate non-cullin proteins in plants. The activity of DenA/1 in *A. nidulans* promotes asexual development and reduces sexual fruiting body formation in light, whereas the deletion of both deneddylases leads to a complete blockage of multicellular fungal development [35,36].

Multicellular fungal development and concomitant secondary metabolism are controlled by the velvet protein family, which consists of four members in *A. nidulans* (VeA, VelB, VelC, VosA), which can form different homo- and heterodimers. The characteristic velvet domain is structurally similar to the mammalian Rel-homology domain (RHD) of human NF-κB proteins, which control human immune and stress signaling pathways. The subcellular localization and the translocation of fungal velvet domain or mammalian RHD domain proteins from the cytoplasm to the nucleus are tightly regulated and essential for their function [37,38,39,40].

Nuclear import, as well as protein stability of the fungal velvet protein VeA, are reduced by velvet interacting proteins as VipC, which can be localized at the cell membrane during sexual development and released to the cytoplasm and transported into the nucleus to support asexual development in an epigenetic signal transduction pathway [41]. VeA enters the nucleus together with VelB, where they form the so-called velvet complex together with the methyltransferase LaeA, which supports sexual development and the appropriate secondary metabolism [37,42]. LaeA influences the expression of secondary metabolite gene clusters, which encode agents with carcinogenic or antibiotic properties like sterigmatocystin or penicillin [42]. Velvet proteins protect the fungus through the regulation of the secondary metabolite profile against stressors. Strains lacking VeA, as well as *csn* deletion strains, are unable to produce sexual fruiting bodies, whereas the overexpression of the *veA* encoding gene leads to cleistothecia formation even in the light, where the sexual developmental cycle is normally repressed [43,44]. So far, there is no experimental evidence that deubiquitinating enzymes regulate the homo- and heterodimer formation of velvet proteins, their subcellular localization or their protein abundance.

The filamentous ascomycete *A. nidulans* carries 22 genes for different putative deubiquitinating enzymes that can be assigned into five different subfamilies. This study characterizes the ubiquitin-specific protease A (UspA). It reveals that the interplay and association of the fungal UspA/Usp15 with the COP9 signalosome fine tunes fungal multicellular development and secondary metabolism. The protein levels of the velvet domain protein VeA are reduced after the early phases of asexual and sexual development presumably through stabilizing the CRL Fbox receptor protein Fbx23.

## 2. Materials and Methods

### 2.1. Strains and Growth Conditions

*Escherichia coli* strains were cultivated in lysogeny broth (LB) medium containing 1% (*w/v*) tryptone, 0.5% (*w/v*) yeast extract, 1% (*w/v*) NaCl [45]. For solid plates, 2% (*w/v*) agar was added. 100 µg/mL ampicillin final concentration was used as a selection marker. *E. coli* strains were cultivated overnight in liquid or on solid media.

*Aspergillus nidulans* strains were grown on minimal medium (MM) containing 1% (*w/v*) glucose, 1x AspA (7 mM KCl, 70 mM NaNO_3_, 11.2 mM KH_2_PO_4_, pH 5.5), 2 mM MgSO_4_, 0.1% (*v/v*) trace element solution (76 µM ZnSO_4_, 178 µM H_3_BO_4_, 25 µM MnCl_2_, 18 µM FeSO_4_, 7.1 µM CoCl_2_, 6.4 µM CuSO_4_, 6.2 µM Na_2_MoO_4_, 174 µM EDTA) pH 5.5 [46]. AGB551 served as wild type background strain if not indicated otherwise [47]. The medium was supplemented according to specific strain requirements with 0.1% (*v/v*) pyridoxine, 5 mM uridine. 2% agar and 0.1% (*w/v*) uracil were added for solid medium. For the selection of strains after transformation, 10 µg/mL phleomycin or 120 ng/mL nourseothricin was added to the medium. A recyclable marker cassette was used [48]. Marker cassette recycling was done on medium containing 0.5% (*w/v*) glucose and 0.5% (*w/v*) xylose as carbon source. *A. nidulans* liquid cultures were grown in flasks with indentations for 20 h at 37 °C on a rotary shaker if not indicated otherwise. Agar plates were incubated for three to five days for fungal growth. The fruiting body formation was analyzed after seven days of growth at 37 °C in darkness.

*S. cerevisiae* strains were grown in yeast extract-peptone-dextrose growth medium (YPED) medium (2% (*w/v*) bactopeptone, 1% (*w/v*) yeast extract, 2% (*w/v*) glucose). For solid plates, 2% (*w/v*) agar was added. Yeast strains were grown after transformation in Synthetic Complete (SC) medium (0.15 % (*w/v*) YNB–aa–as (yeast nitrogen base w/o amino acids and ammonium sulfate), 0.5% (*w/v*) ammonium sulfate, 0.2 mM inositol, 0.2% (*w/v*) amino acid powder mix, and 2% (*w/v*) raffinose), lacking histidine, tryptophan, and uracil. Liquid yeast strains were cultivated overnight, whereas agar plates were incubated for two to three days at 30 °C.

### 2.2. Plasmid and Strain Construction

All plasmids constructed in this study were propagated in *E. coli* DH5α and extracted with the QIAprep^®^ Spin Miniprep Kit (Qiagen, Hilden, Germany) or the NucleoSpin^®^ Plasmid Kit (Macherey-Nagel, Düren, Germany). All constructed plasmids were sequenced by Seqlab Sequence Laboratories GmbH. Plasmid maps were created with the Lasergene software package (DNA Star Inc., Madison, WI, USA). The correct integration of the constructs into the genome of *A. nidulans* was verified by Southern hybridization experiments. Therefore, the AlkPhos Direct Labeling and Detection System (GE Healthcare, Chicago, IL, USA) was used according to the manufacturer’s instructions. *A. nidulans* strains transformed with the plasmid pME3857 were verified only by fluorescence.

#### 2.2.1. Construction of Cloning Vector pME4696

The plasmid pBluescript KS was amplified with primers flip-1 and flip-2, which contained a PmlI or SwaI restriction site in their overhang, respectively. The linear PCR fragment and the SfiI digested nourseothricin recyclable marker cassette (natRM) from plasmid pME4304 were ligated in a Seamless cloning reaction. The resulting plasmid served as a cloning vector for further constructs.

#### 2.2.2. Construction of pME4701 Resulting in Strains AGB1169 and AGB1233

Plasmid pME4654 carries the deletion cassette of *csnE* [28] and was used for the construction of *A. nidulans* strain AGB1169. Restriction digest of pME4654 with PmeI and the transformation of the linear fragment into AGB551 resulted in strain AGB1169. The *csnE* deletion was complemented with plasmid pME4701. The 5′ flanking region, together with the *csnE* ORF, was amplified from the genomic DNA with the primers AL39/CM99. For the amplification of the 3′ flanking region, AL47/AL48 were used. The recyclable marker cassette used for this cloning reaction was excised with SfiI from pME4304. All fragments were cloned together in a GeneArt Seamless Cloning and Assembly Kit (Invitrogen, Carlsbad, CA, USA), reactions performed according to the manufacturer’s instructions. The complementation construct was excised with PmeI and transformed as a linear fragment into AGB1169, resulting in strain AGB1233.

#### 2.2.3. Construction of Plasmids pME4703 and pME4704 and Resulting Strains AGB1156 and AGB1157

For the construction of the *uspA* deletion cassette, the 5′ flanking region was amplified with the primer pair SI29/SI30, which contain a BstEII restriction site in their overhangs. The *pyroA4* marker was derived from *Aspergillus fumigatus* and was amplified with the primer pair SI27/SI28, whereby SI27 encompasses a BstEII restriction site, and SI28 contains a NdeI restriction site. The 3′ flanking region was amplified with primers SI31/SI32 that contain NdeI restriction sites in their overhangs. After successful subcloning of these three fragments into the pBlueScript cloning vector for the *pyroA* marker and into a pJET vector for the flanking regions, restriction digestion with BstEII and NdeI and subsequent ligation reactions resulted in the plasmid pME4703. For transformation into *A. nidulans* AGB551, the plasmid was digested with BamHI and XhoI to integrate the linear deletion cassette into the original *uspA* gene locus, leading to the strain AGB1156.

For the complementation plasmid pME4704, the genomic locus of *uspA* was amplified with SI29/SI32 from wild type genomic DNA and ligated into the EcoRV restriction site of pME3281. The circular plasmid was transformed into AGB1156, resulting in ectopic complementation of the *uspA* deletion, whereas the original gene locus still contains the *pyroA4* marker. The resulting strain is AGB1157.

#### 2.2.4. Construction of Plasmid pME4706 and *A. nidulans* Strain AGB1159 and AGB1161

The GeneArt Seamless Cloning and Assembly Kit (Invitrogen, Carlsbad, CA, USA) was used for the preparation of plasmid pME4706 according to the manufacturer’s instructions. Therefore, the 5′ flanking region of *uspA* together with the *uspA* ORF was amplified from genomic DNA of the wild type AGB551 with primers CM37/CM48. The PreScission cutting site, the linker, as well as the *gfp,* were amplified together with primers AMK82/AMK85 from pME4722 [28]. A SwaI cutting site is included in the overhang of primer AMK85, which allows the cloning in two steps. The two described fragments were cloned in a Seamless Cloning reaction into the linearized pUC19 vector. In a second step, the resulting pre-plasmid was digested with SwaI, and the two remaining fragments were cloned into it. The *gpdA* promoter and the *nat* gene were amplified from pME4304 with primers AMK86/CM42, and the remaining 3′ flanking region of *uspA* was amplified from genomic DNA of AGB551 with the primer pair CM43/CM44. Primers CM37 and CM44 encompassed a PmeI restriction site in their overhangs. Hence, the construct was excised with PmeI from the plasmid backbone, and the linearized fragment was transformed into AGB551, resulting in the strain AGB1159. The, *A. nidulans* strain AGB1161 resulted from the transformation of AGB1159 with pME3857.

#### 2.2.5. Construction of Plasmid pME4707 and *A. nidulans* Strain AGB1162 and AGB1163

The plasmid pME4707 contains the DNA coding for an UspA-GFP fusion protein with two point mutations in the *uspA* ORF. The cysteine residue 469 and the cysteine 1066 were mutated to alanine, resulting in the so-called *uspA^AA^* mutation. Therefore, *uspA*^1-1414^ was amplified with primer CM138/CM139, whereas CM138 contained in its overhang a PmeI cutting site. *uspA*^1400-3250^ was amplified with CM140/CM141. The last fragment was amplified with primers CM142/EB2. The primer CM139/CM140 contained a mutated triplet (TGC to GCC), which mutates C469 to alanine and CM141/CM142 contained the mutation of C1066 to alanine (TGC to GCC). The plasmid pME4706 served as a template for these PCRs. All fragments were fused through fusion PCRs and ligated into the PmlI restriction site of pME4696. The 3′ flanking region of *uspA* was amplified from wild type genomic DNA with the primer pair CM43/CM44 and ligated into the SwaI site of pME4696. The primer CM44 contains a PmeI restriction site in its overhang, and therewith the linear construct was excised and transformed into AGB551, resulting in AGB1162. The, *A. nidulans* strain AGB1163 resulted from the transformation of AGB1162 with pME3857.

#### 2.2.6. Construction of BiFC Plasmids pME4708-4712 and the Resulting, *A. nidulans* Strains AGB1170-AGB1174

For the construction of BiFC plasmids, the plasmid pME4313 was used as a backbone, which contains a bidirectional nitrate promoter that is flanked by a SwaI and PmeI restriction sites. All BiFC plasmids were prepared with the T4 DNA Ligase (Thermo Fisher Scientific, Waltham, MA, USA) during an overnight ligation reaction at 16 °C.

For the plasmid pME4708, the C-terminal part of YFP (*cYFP*) was amplified from plasmid pME4601 with the primer pair AMK168b/AMK169b and fused to *uspA*, which was amplified with CM161/CM2 through fusion-PCR. The primer AMK169b contained a 24 bp long linker, which is located between *cYFP* and *uspA*. The 4628 bp long fusion product was ligated into the SwaI restriction site of pME4313. Furthermore, the ORF of *csnB* was amplified from the genomic DNA of AGB551 with the primer pair CM163/CM164 and fused through PCR to the N-terminal half of YFP (*nYFP*), which was amplified with primers AMK163/CM94 from pME4685 [28]. This fusion product was ligated into the PmeI restriction site and resulted in the plasmid pME4708, which was transformed into AGB1014 and resulted in the strain AGB1170.

The plasmid pME4709 encompasses the *nYFP:csnB* fusion in the PmeI restriction site of pME4313. Only the *cYFP* was amplified with AMK168b/CM165 from pME4601 and ligated into the SwaI restriction site. The transformation of this plasmid into AGB1014 resulted in AGB1171.

Plasmid pME4710 encompasses the *cYFP:uspA* fusion in its SwaI restriction site, whereas into the PmeI restriction site, the empty *nYFP* was ligated that was amplified with AMK163/CM162 from pME4601. The transformation of pME4710 into AGB1014 resulted in the *A. nidulans* strain AGB1172.

The plasmid pME4711 contained the *cYFP:uspA* fusion in its SwaI restriction site. Furthermore, *csnF* was amplified from the genomic DNA of AGB551 with the primer pair CM166/CM167 and fused N-terminally to *nYFP* that was amplified with AMK163/CM94 from pME4685 [28]. Both fragments were fused through a PCR and ligated into the PmeI restriction site. Plasmid pME4711 was transformed into AGB1014, and this resulted in the strain AGB1173.

For the construction of pME4712, the *nYFP:csnF* fusion was ligated into the PmeI restriction site of pME4313 and the empty *cYFP* fragment into the SwaI restriction site. The transformation of pME4712 into AGB1014 resulted in AGB1174.

#### 2.2.7. Construction of Plasmid pME4714 and the *A. nidulans* Strains AGB1165 and AGB1167

The plasmid pME4714 was cloned with the GeneArt Seamless Cloning and Assembly Kit (Invitrogen, Carlsbad, CA, USA) and used according to the manufacturer’s instructions. Therefore, the 5′ flanking region, together with the *veA* ORF, was amplified with KT197/SR44. Thereby, KT197 contains a *Pme*I cutting site. The linker-GFP fragment was amplified with SR18/SR20. The 3′ flanking region of *veA* was amplified with KT142/KT198, whereby KT198 also contains a PmeI cutting site. The recyclable marker cassette was digested out of pME4304 with SfiI and used for the cloning reaction. The PmeI digested linear fragment was transformed into AGB1066 and resulted in the strain AGB1165. The, *A. nidulans* strain AGB1167 was constructed by the transformation of pME3857 into AGB1165.

#### 2.2.8. Construction of AGB1164, AGB1166, AGB1234, and AGB822

The plasmid pME4714 was digested with PmeI, and the linearized fragment was transformed into AGB1156. This resulted in the *A. nidulans* strain AGB1164. The transformation of pME3857 into AGB1164 resulted in AGB1166. The, *A. nidulans* strain AGB1234 resulted out of the transformation of the pME4714 digested PmeI fragment into AGB822. AGB822 resulted from the replacement of the *fbx23* ORF with the *pyrG* marker from *A. fumigatus* through homologous recombination. The 5′ and 3′ flanking regions of *fbx23* were fused through PCR with the *pyrG* marker sequence. The fusion-PCR product was used for transformation into *A. nidulans* in AGB551. Positive clones were screened with PCR and verified with Southern hybridization.

#### 2.2.9. Construction of Plasmid pME4715

For the construction of pME4715, *uspA* was amplified from cDNA with primers CM128/CM129, which was cloned into the NotI restriction site of pME3229. The circular plasmid was transformed into the yeast strain EGY48.

### 2.3. Transformations of E. coli, A. nidulans, and S. cerevisiae

The, *E.coli* strain DH5α was used for transformations that were performed like described previously [49,50]. *A. nidulans* transformations were performed as described elsewhere [51]. The protoplastation was initiated with 15 mL 30 mg/mL Vinoflow^®^ Max or Vinotaste^®^ Pro from Novozymes (Bagsvaerd, Denmark) and 15 mg/mL lysozyme from Serva (Heidelberg, Germany) dissolved in citrate buffer (150 mM KCl, 580 mM NaCl, 50 mM Na-citrate, pH 5.5) for 105 min at 30 °C. Strains were verified by Southern hybridization [52]. Transformations of *S. cerevisiae* strain EGY48 were performed with the LiAc/SS Carrier DNA/PEG method [53,54]. Plasmids used for the transformations are listed in Table S1, and resulting strains used or prepared in this study are listed in Table S2. All oligonucleotides used for the construction of plasmids are given in Table S3, and oligonucleotides used for qRT experiments are given in Table S4.

### 2.4. Genomic DNA Extraction

*A. nidulans* strains were grown overnight at 37 °C in a liquid medium. Mycelium was harvested with a miracloth filter and grained manually in liquid nitrogen. For cell lysis, 500 µL of gDNA extraction buffer (200 mM Tris-HCl pH 8.5, 250 mM NaCl, 25 mM EDTA, 0.5% (*w/v*) SDS, recipe modified from [55]) was used, and gDNA isolation performed like described previously [48]. Briefly, 500 μL of the extraction buffer was mixed with the mycelium and incubated for at least 15 min at 65 °C. After cooling the samples on ice for 5 min, 100 µL 8 M potassium acetate was added, and the tube was inverted six to eight times before the centrifugation step at 13,000 rpm for 15 min. The precipitation step of cell debris and proteins with potassium acetate was repeated once. Afterward, the supernatant was transferred into a fresh reaction tube, and the DNA was precipitated by the addition of 300 µL isopropanol and another centrifugation step. The precipitated DNA was washed with 100% (*v/v*) ethanol and dried afterward at 42 °C. The DNA was dissolved in 100 µL ddH_2_O.

### 2.5. Database Searches and BLAST Analyses

Text-based searches for gene loci that express proteins with ubiquitin-specific protease activity was performed in the AspGD and FungiDB database [56,57]. These protein sequences were blasted with the NCBI protein basic local alignment search tool (BLAST) using the Reference proteins (RefSeq) databases of *S. cerevisiae* (TaxID: 4932) or *H. sapiens* (TaxID: 9606) [58]. Resulting proteins with the lowest *E*-value were considered as closest yeast or human DUB to the respective *A. nidulans* protein. Domain predictions for uncharacterized proteins were performed with the NCBI conserved domain (CD) search [59].

### 2.6. Phenotypical Characterization

The quantification of conidiospores was performed after three and five days of growth of *A. nidulans* colonies at 37 °C during constant illumination. Therefore, 5000 spores of the respective strains were point-inoculated on MM supplemented with 0.1% (*v/v*) pyridoxine, 5 mM uridine, and 0.1% (*w/v*) uracil. Spores were counted with Coulter Z2 particle counter (Beckman Coulter GmbH). For the analyses of sexual fruiting body formation, 5000 spores were either point-inoculated and incubated for seven days in darkness at 37 °C, or 30,000 spores were equally distributed on an agar plate and incubated for three, five, or seven days in darkness. Pictures of cleistothecia were taken with an Axiolab microscope (Carl Zeiss Microscopy, Oberkochen, Germany) and photomicrographs with the SZX12-ILLB2-200 binocular microscope (Olympus, Shinjuku, Japan).

### 2.7. Protein Isolation and Western Blot

Strains were grown for 20 h at 37 °C on a rotary shaker and either harvested, washed with 0.96% NaCl solution, and frozen in liquid nitrogen or the mycelia were shifted on a solid agar plate containing MM. The plates were incubated either in light to induce asexual development or in darkness for the induction of sexual development for up to 24 h or 48 h, respectively. If required, samples of the mycelium were taken every six hours. For the extraction of proteins, the mycelium was grained manually and dissolved in buffer B* (300 mM NaCl, 10 mM Tris-HCl pH 7.5, 0. 5 mM EDTA, 0.05% (*v/v*) NP-40, 10% (*v/v*) glycerol), which was freshly supplemented with 1 mM PMSF, 1.5 mM DTT, 1 tablet/50 mL complete EDTA-free protease inhibitor cocktail (Roche, Basel, Switzerland). The samples were centrifuged for 30 min at 13,000 rpm at 4 °C. The supernatant was transferred into a new reaction tube, and centrifugation was repeated for 10 min. The concentration was determined with a NanoDrop ND-1000 spectrophotometer (Peqlab, Erlangen, Germany). Samples were adjusted to the same final concentration, and 20 µL protein sample buffer (250 mM Tris-HCl pH6.8, 15% (*v/v*) β-mercaptoethanol, 30% (*v/v*) glycerol, 7% (*v/v*) SDS, 0.3% (*w/v*) bromophenol blue) was added to 40 µL protein extract and boiled at 95 °C for 10 min.

Equal amounts of proteins were loaded on a 12% SDS gel (stacking gel: 3.67 mL H_2_O, 625 µL 1 M Tris pH 6.8, 30 µL 10% (*w/v*) SDS, 650 µL 30% (*v/v*) acrylamide, 5 µL TEMED, 25 µL 10% (*w/v*) APS), separation gel (2.8 mL H_2_O, 3.75 mL 1 M Tris pH 8.8, 100 µL 10% (*w/v*) SDS, 3.3 mL 30% (*v/v*) acrylamide, 10 µL TEMED, 50 µL 10% (*w/v*) APS). Separated proteins were blotted onto a nitrocellulose membrane (Amersham^TM^ Protran^TM^ 0.45 µm NC nitrocellulose membranes, GE Healthcare) and blocked with 5% (*w/v*) milk powder solution. As primary antibodies, αGFP (sc-9996, Santa Cruz Biotechnology, Dallas, TX, USA) or αUbiquitin (clone P4D1-A11, Merck Millipore, Darmstadt, Germany) were used in a 1:500 or 1:2000 dilution, respectively, in 5% milk powder/TBST solution. As secondary antibodies, αRabbit (G21234, Invitrogen, Carlsbad, CA, USA) or αMouse (115-035-003, Jackson Immuno Research, West Grove, CA, USA) were used in a 1:1000 dilution. The detection of the chemiluminescent signals was done with a 1:1 mixture of solution A (2.5 µM luminol, 400 µM paracoumarat, 100 mM Tris-HCl pH 8.5) and solution B (5.4 mM H_2_O_2_, 100 mM Tris-HCl pH 8.5) that were incubated on the membrane for 90 s in darkness. Signals were detected with a Fusion-SL7 chemiluminescence detection system (Peqlab Erlangen, Germany), and pictures were recorded with the Fusion 15.15 software (Vilber Lourmat, Eberhardzeil, Germany). Staining the membrane prior to blocking and antibody reactions with Ponceau S solution served as a loading control and were used for normalization. The quantification of the pixel intensity was done with Bio-1D software (Vilber Lourmat, Eberhardzeil, Germany).

### 2.8. RNA Extraction and Quantitative Real-Time PCR

Strains were inoculated with 10^6^ spores/mL and grown for 20 h in submerged culture for analyzing gene expression during vegetative growth. The mycelium was shifted onto agar plates and further incubated at 37 °C in light (for the induction of asexual development) or in darkness (for the induction of sexual development) for 24 or 48 h. Mycelium was grained, and RNA was extracted using the RNeasy Plant Miniprep Kit (Qiagen, Hilden, Germany) according to the manufacturer’s instructions. The RNA concentration was determined using the NanoDrop ND-1000 spectrophotometer (Peqlab, Erlangen, Germany). cDNA was prepared with the QuantiTect Reverse Transcription Kit (Qiagen, Hilden, Germany) according to the manufacturer’s instructions using 0.8 µg RNA.

Quantitative real-time PCR analyses were performed to determine gene expression levels. Therefore, the MESA GREEN qPCR MasterMix Plus for SYBR Assay (Eurogentec, Lüttich, Germany) was used. The measurements were done with the CFX Connect Real-Time System (BioRad, Hercules, CA, USA) and analyzed with the respective CFX Manager 3.1 software (BioRad, Hercules, CA, USA). Significances were calculated with this program as well, and the regulation threshold was set to 2. *15S rRNA* and *h2A* served as the reference genes.

### 2.9. Yeast-Two-Hybrid Assay

Yeast-two-hybrid (Y2H) protein interaction studies were performed as described previously [60,61]. The interaction of UspA with the single CSN subunits was analyzed. Thereby, *uspA* cDNA was cloned into the NotI restriction site of pEG202 (pME3229). The single CSN subunit encoding genes were cloned into the pJG4-5 (pME3230) prey vector, previously [24]. The bait and one of the prey plasmids, respectively, were transformed into EGY48 [61]. This yeast strain contains the *LEU2* reporter system. Positive clones were inoculated in SC medium (0.15% (*w/v*) YNB-aa-as (yeast nitrogen base w/o amino acids and ammonium sulfate), 0.5% (*w/v*) ammonium sulfate, 0.2 mM inositol, 0.2% (*w/v*) amino acid powder mix, and 2% (*w/v*) raffinose), lacking histidine, tryptophan, and uracil and incubated on rotary shaker at 30 °C until they reached an OD_600_ of 0.1. Interacting bait and prey proteins induce the expression of the *LEU2* reporter gene in the presence of galactose as a carbon source. Different dilutions from OD_600_ of 0.1 to 0.00001 of yeast cells were used for the experiment and spotted on different media. Agar plates containing SC medium with 2% (*w/v*) glucose as carbon source and supplemented with 2% (*w/v*) leucine served as growth control. Plates lacking the leucine supplementation served as a negative control. Furthermore, agar plates containing 2% (*w/v*) galactose and 1% (*w/v*) raffinose as a carbon source while lacking leucine enabled the growth of the strains expressing interacting bait and prey proteins. The plates were incubated for three to five days at 30 °C.

### 2.10. Fluorescence Microscopy

*A. nidulans* strains were inoculated in liquid minimal media on sterile cover slides (18 × 18 mm, Th. Geyer, Höxter, Germany) that were placed in sterile petri dishes. In 400 µL medium, 2,000 spores were inoculated and grown for 20 h at 37 °C under illumination. Just before microscopy, the remaining medium was removed from the cover slide with a tissue. Twenty microliters of the fresh minimal medium were placed on an object slide, and the cover slide was placed on this fresh medium. The cover slide was fixed with nail polish. The inverted confocal microscope Zeiss AxioObserver, Z.1, which is equipped with Plan-Neofluar 63x/0.75 (air), Plan-Apochromat 63x/1.4 oil, Plan-Apochromat 100x/1.4 oil objectives (Zeiss, Oberkochen, Germany), and a QuantEM:512SC camera (Photometrics, Tucson, AZ, USA), was used in combination with the SlideBook 6.0 software package (Intelligent Imaging Innovations GmbH, Göttingen, Germany). Thereby, pictures were only taken with the Plan-Apochromat 100x/1.4 oil objectives (Zeiss, Oberkochen, Germany). The wild type strain AGB551 or the AGB1014, which expresses *^P^gpdA:rfp:h2A*, was included in the analyses for the normalization of GFP fluorescence.

### 2.11. GFP Pull-Down

The strains used for GFP pull-down experiments were inoculated with 10^6^ spores/mL and grown for 20 h in submerged cultures at 37 °C. After harvesting, the mycelia were washed with 0.96% (*w/v*) NaCl solution that was supplemented with 1 mM PMSF and 1% (*v/v*) DMSO. The mycelium was manually grained, and equal amounts of grained mycelium of each strain were used for the GFP Trap experiment. The mycelium was dissolved in buffer B* (freshly supplemented with 1.5 mM DTT, 1 tablet/50 mL complete EDTA-free protease inhibitor cocktail (Roche, Basel, Switzerland), 1 mM PMSF, phosphatase inhibitor mix (1 mM NaF, 8 mM β-glycerolphosphate disodium pentahydrate, 0.5 mM sodium-orthovanadate)) and centrifuged for 1 h with 15,000 rpm at 4 °C. The supernatant was filtered again through 0.2 µm filters and afterward incubated with buffer B* pre-equilibrated GFP-Trap^®^_A beads (Chromotek, Planegg-Martinsried, Germany) for 2 h on a rotator at 4 °C. For 5 mL of the protein containing supernatant, 60 µL of GFP beads were used. The incubation was done in polypropylene columns, which allowed the protein-containing supernatant after the incubation time to pass through the column, whereas the beads together with the bound proteins stayed inside the column. The beads were washed with 1.5 mL buffer I (300 mM NaCl, 10 mM Tris pH 7.5, 0.5 mM EDTA) and 1 mL buffer II (500 mM NaCl, 10 mM Tris pH 7.5, 0.5 mM EDTA), which were both freshly supplemented with 1 mM PMSF, 1.5 mM DTT, 1 tablet/50 mL complete EDTA-free protease inhibitor cocktail. The elution of the bound proteins was performed with 150 µL 0.2 M glycine pH 2.5, which was mixed for 30 s with the beads. Afterward, the solution was collected into a LoBind reaction tube that contained 15 µL Tris pH 10.4 to neutralize the pH. The elution step was performed three times, and the concentration was determined with the NanoDrop ND-1000 photospectrometer (Peqlab, Erlangen, Germany). The elutions were either boiled with protein sample buffer at 95 °C for loading on SDS PAGE or precipitated with chloroform/methanol extraction for in-solution tryptic digestion.

### 2.12. Tryptic Digestion

The in-gel tryptic digestion of proteins was performed as described earlier [62]. Prior to in-solution tryptic digestion, proteins were precipitated according to Wessel and Flügge [63]. Therefore, 400 µL methanol was added to 100 µL of the protein sample. The solution was mixed and centrifuged for 10 s at 10,000 rpm before 100 µL of chloroform was added, and the mixing and centrifugation steps were repeated. Three-hundred microliters ddH_2_O were added, and the solution was vortexed for 30 s. The following centrifugation step for 3 min at 4 °C at 10,000 rpm resulted in phase separation, with the proteins in the interphase. The aqueous, upper phase was carefully removed. As a final step, 300 µL methanol was added to the lower phase, and centrifugation for 10 min at 4 °C and 13,000 rpm resulted in the formation of a protein pellet. The supernatant was discarded, and the protein pellet was dried overnight at room temperature. The protein pellet was dissolved in a 0.1% (*w/v*) RapiGest^TM^ SF solution (Waters GmbH, Milford, MA, USA) and used according to the manufacturer’s instructions. The Sequencing Grade modified Trypsin from Promega (Fitchburg, MA, USA) was used for in-gel and in-solution digestion. After digestion of proteins, the Stop and Go Extraction (Stage) Tip Purification was performed [64]. For LC-MS/MS analysis, peptides were dissolved in 20 µL sample buffer (98% (*v/v*) ddH_2_O, 2% (*v/v*) acetonitrile, and 0.1% (*v/v*) formic acid).

### 2.13. LC-MS Analysis

An Orbitrap Velos Pro mass spectrometer (Thermo Fisher Scientific, Waltham, MA, USA) and an RSLCnano Ultimate 3000 chromatography system (Thermo Fisher Scientific, Waltham, MA, USA) were used for LC-MS analysis of GFP pull-down elution fractions. A water-acetonitrile gradient was used for the separation of peptides on a reverse phase chromatography on an Acclaim PepMap RSLC column (Thermo Fisher Scientific, Waltham, MA, USA). Online ionization of peptides by nano-electrospray at 2.4 kV was done utilizing the Nanospray Flex Ion Source (Thermo Fisher Scientific, Waltham, MA, USA). Acquisition of full scans with a mass range of 300–1850 *m*/*z* was done with the Orbitrap-FT analyzer at a resolution of 30,000. The LTQ Velos Pro ion trap was used for the collision-induced dissociation (CID) fragmentation of data-dependent top-ten peptides with dynamic exclusion of precursor masses enabled. XCalibur 2.2™ software (Thermo Fisher Scientific, Waltham, MA, USA) was used for the acquisition of mass spectra and programming of the LC-MS method. Downstream data analysis was performed with the MaxQuant and Perseus software package [65,66]. Proteins also identified from the control sample and proteins belonging to the categories “Potential Contaminants”, “Reverse”, and “Only identified by site” were not considered further during the analyses. Proteins identified at least two biological replicates with at least two unique peptides and alog_2_ label-free quantification (LFQ) of >20 were considered as putative interaction partners.

## 3. Results

### 3.1. The Repertoire of Deubiquitinating Enzymes (DUBs) in Aspergillus nidulans

Deubiquitinating enzymes, which remove ubiquitin molecules or chains from target proteins [67], are not well studied in Aspergilli. Text-based database analyses were applied using AspGD [56] and FungiDB [57]. BLAST analyses [58] allowed the classification of the putative DUBs into different subfamilies.

In total, 22 genes encoding putative deubiquitinating enzymes in *A. nidulans* were identified and assigned to five different subfamilies (Table 1). The largest family is built by the ubiquitin-specific proteases (USPs) like in animals or yeast and includes CreB as only so far characterized DUB of *A. nidulans* [17,18]. The group of ubiquitin C-terminal hydrolases (UCH) encompasses four members, and the groups of orthologs to the ovarian-tumor proteases (OTUs) as well as JAB1/MPN/Mov34 metalloprotease DUBs (JAMM) comprise two members each. The motif interacting with Ub-containing novel DUB family (MINDY) is represented by only one protein in *A. nidulans* (Table 1), whereas representatives of the families of Machado-Josephin domain proteases and the Zinc finger with UFM1-specific peptidase domain proteins (ZUFSP), which are present in higher eukaryotes, were not identified in *A. nidulans* [14,67,68].

In this study, we focus on UspA as an ortholog of human Usp15, which forms together with Usp4 and Usp11 a subfamily. All three mammalian proteins have a similar protein structure and comparably highly conserved domain architecture [69]. Fungal UspA also shares sequence similarities with human Usp4 and Usp11. The *A. nidulans uspA* gene is localized on chromosome I and consists of an open reading frame of 4348 base pairs including two introns. The deduced 1418 amino acid UspA protein of 156.6 kDa is characterized by a domain specific for ubiquitin-specific proteases (DUSP) near the N-terminus and an ubiquitin carboxyl-terminal hydrolase domain, which is specific for ubiquitin-specific proteases (USP). This catalytic domain carries all residues that are important for catalytic activity, which is highly conserved among fungi and up to humans (Figure S1).

### 3.2. CsnE Causes the Repression of Ubiquitin-Specific Protease Encoding Genes

The COP9 signalosome of *A. nidulans* consists of eight subunits (CsnA-CsnH) [24]. A pre-assembled seven-subunit pre-CSN finally incorporates the catalytically active subunit CsnE [22,26] to change cullin E3 RING ligase activities by removing Nedd8 [70,71]. In a *csnE* deletion strain, neddylated (active) CullinA accumulates as the deneddylation reaction is impaired [26]. Therefore, the number of ubiquitinated proteins might increase in these strains.

The total amount of ubiquitinated proteins in the fungal cell was compared in wild type, *csnE* deletion, and complementation strains, which were grown in submerged cultures and afterward transferred on solid agar plates to initiate asexual (24 h light) or sexual (48 h darkness) development. Western blot experiments with total cellular crude extracts from the different developmental stages were performed. The number of ubiquitinated proteins was not altered in a *csnE* deletion strain compared to wild type or complementation (Figure S2).

**Figure 1:**
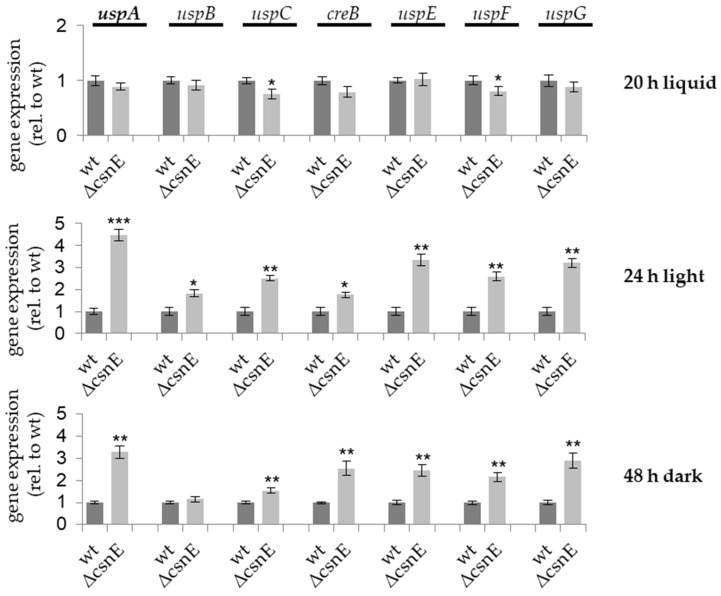
The expression of *usp* encoding genes depends on CsnE during the multicellular development of *A. nidulans*. Fungal strains were grown for 20 h in liquid cultures and mycelia were harvested or afterwards shifted onto solid agar plates and incubated for 24 h in light to initiate asexual development or for 48 h in darkness to initiate sexual development at 37 °C, respectively. RNA was isolated from these samples and cDNA was transcribed. qRT-PCRs were performed with *h2A* and *15s rRNA* as reference genes. Wild type expression was set to one. Standard deviations are shown that derive from up to four biological replicates with three technical replicates each (*** *p* ≤ 0.001; ** *p* ≤ 0.01, * *p* ≤ 0.05).

The same developmental time points were used to analyze the expression levels of different USPs (*uspA*, *uspB*, *uspC*, *creB*, *uspE*, *uspF,* and *uspG*) in a *csnE* deletion strain compared to wild type. Increased transcription was observed for all tested deubiquitinase encoding genes during at least one developmental stage. *uspB* is the only gene, which shows increased transcript levels only during asexual development, whereas six *usp* genes were de-repressed during asexual as well as during sexual development. The expression of *uspA* was more than four times upregulated during asexual development in the *csnE* deletion strain compared to wild type and thereby shows the highest regulation of all *usp* encoding genes that were included in this study (Figure 1).

Dysfunction of the COP9 signalosome in *A. nidulans* caused by deletion of the gene encoding CsnE does not lead to changes in the total ubiquitination pattern of proteins during the whole fungal development, but to the upregulation of genes encoding USPs. This indicates a complex interplay between ubiquitination and deubiquitination already on the transcriptional level mediated by the COP9 signalosome.

### 3.3. UspA-GFP Interacts with CSN Subunits In Vivo and In Vitro and is Mainly Localized in the Periphery of Nuclei

**Figure 2:**
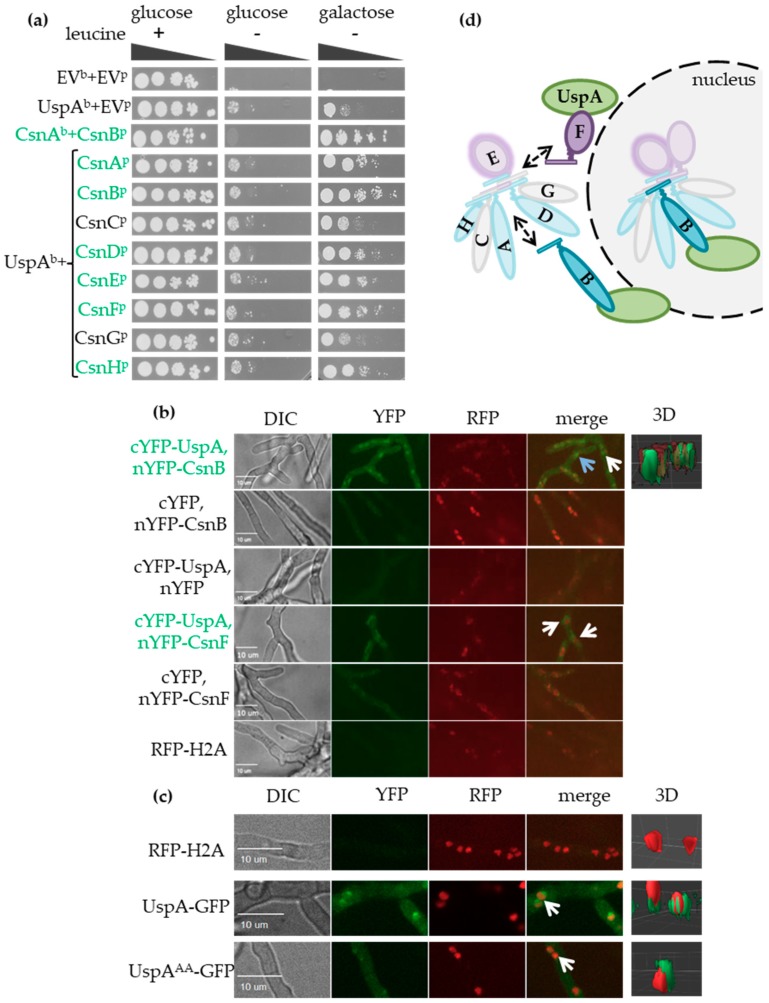
UspA-GFP is localized close to nuclei and interacts with COP9 signalosome subunits in vitro and in vivo*.* (**a**) Growth analysis of yeast cells, expressing UspA as bait (^b^) from the constitutive *ADH1* promoter, and CSN subunits as prey (^p^), driven by the inducible *GAL1* promoter on non-inducing (glucose) and inducing (galactose) medium. Yeast strains containing empty vectors (EV) served as control. Left panel: cells were spotted on selection medium containing glucose and leucine (positive growth control). The medium used for strain cultivation shown in the middle panel is not supplemented with leucine, which serves as negative growth control. The observed growth is due to auto-activation of the promoter due to the presence of *uspA* as bait. Yeast cells growing on the selective interaction medium (right panel) contain interacting bait and prey proteins. (**b**) BiFC assay was performed with CsnB and UspA, as well as with CsnF and UspA, that are fused to one half of YFP, respectively, during vegetative development in *A. nidulans* hyphae. Therefore, 2000 spores were inoculated in minimal medium and grown for 20 h in light at 37 °C. In the left panel, differential interference contrast (DIC) images of the hyphae are shown. All strains contain RFP tagged H2A to visualize nuclei (red). YFP fluorescent signal indicates the interaction of the two proteins in the nucleus (highlighted with blue arrow) or close to nuclei (highlighted with white arrows). Scale bars represent 10 μm. (**c**) Fluorescence microscopy was performed under the conditions described in (b) to investigate the subcellular localization of UspA-GFP and UspA^AA^-GFP fusion proteins in *A. nidulans* hyphae. (**d**) Model of the localization of UspA and interaction of the DUB with the COP9 signalosome. Subunits with interaction are shown in blue. UspA interacts with CsnB and CsnF in BiFC experiments and with CsnA, B, D, E, F, and H in a Y2H system.

We investigated whether *A. nidulans* UspA interacts with the COP9 signalosome as it is described for the orthologous proteins in other organisms, including humans [19,20]. A yeast-two-hybrid (Y2H) binary protein-protein interaction analysis was performed by fusing the gene encoding UspA to a GAL4-DNA binding domain (bait) and single CSN subunit encoding genes to a GAL4-activation domain (prey) to activate an appropriate *LEU2* reporter construct [60,61]. Interaction of UspA with the CSN subunits was monitored as growth on selective medium lacking leucine as a consequence of *LEU2* reporter activation (Figure 2a, right panel). This Y2H experiment revealed that *A. nidulans* UspA could interact directly with the CSN. The binding surface presumably includes CsnA, B, D, E, F, and CsnH and, therefore, six of the eight CSN subunits, including the catalytically active subunit CsnE. The structure of the human COP9 signalosome suggests that UspA could interact with the helical bundle formed by the C-terminal α-helices of all CSN subunits [22]. The α-helices of the helical bundle of these six UspA interacting subunits protrude into another direction than the missing Csn3 and Csn7 corresponding to CsnC and CsnG [22]. CSN also physically binds and destabilizes the second fungal deneddylase DenA, which supports asexual development. The UspA-CSN and the DenA-CSN interaction surfaces seem to overlap but are presumably not identical, because DenA is destabilized by CsnC and CsnH and in addition by CsnE, F, G and also binds to CsnG and CsnA, E, F in a Y2H analysis [35,72].

The interaction of UspA and CSN subunits was localized in undifferentiated *A. nidulans* hyphae monitored by bimolecular fluorescence complementation (BiFC) experiments (Figure 2b, left panel). The PCI domain containing subunit CsnB and the MPN domain-containing subunit CsnF might be part of different COP9 signalosome subcomplexes [73]. The interaction of UspA and CsnB takes place in the nucleus and outside in close proximity to the nucleus. UspA and CsnF interact mainly in the surrounding of nuclei in the cytoplasm (Figure 2b, right panel: 3D depiction of nuclei in red and the UspA-CsnB interaction in green). In previous studies with *S. pombe*, *S. cerevisiae*, plant, or mammalian cells, the functional COP9 signalosome was mainly found to be located inside nuclei [74,75,76,77].

A functional UspA-GFP fusion protein was expressed under the control of the native *uspA* promoter to investigate the subcellular localization of the DUB itself with fluorescence microscopy. UspA-GFP was located at the nuclear periphery outside of the nucleus, and only a smaller subpopulation was located inside nuclei (Figure 2c). An inactive UspA-GFP protein was expressed to analyze whether activity affects the cellular location. Highly conserved cysteines C469 as part of the catalytic triad and C1066 as part of a zinc finger motif were mutated to alanine (A) codons (Figure S1). The resulting UspA^AA^-GFP variant protein showed similar localization mainly close to nuclei like UspA-GFP, suggesting that cellular localization is independent of enzymatic function (Figure 2c).

A subpopulation of UspA-GFP, as well as UspA^AA^-GFP, resides in the nucleus, where it interacts according to the BiFC experiments with subunits of the COP9 signalosome. This supports the hypothesis that UspA interacts with CSN subcomplexes inside and in close proximity to the nucleus and could play a role in the nuclear/cytoplasmic shuttling of CSN subunits or subcomplexes (Figure 2d).

### 3.4. UspA-GFP Pulls Proteins Related to Nuclear Transport, Transcriptional Processing, and Ubiquitin-Proteasome System

**Figure 3:**
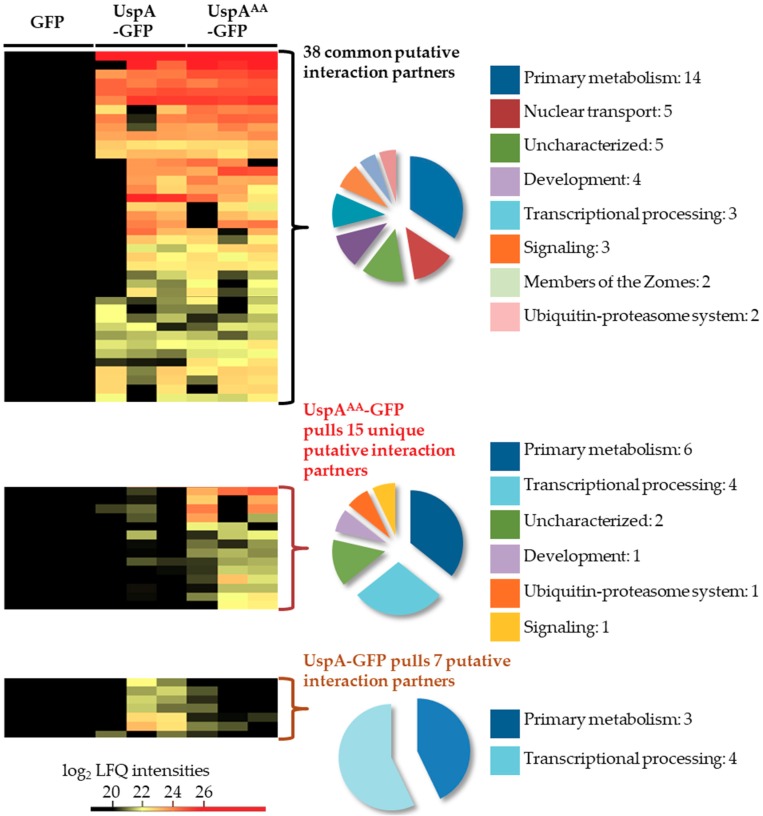
UspA-GFP pull-downs captured proteins related to primary metabolism, nuclear transport, and transcriptional processing. GFP pull-down experiments were performed with *A. nidulans* strains expressing either functional UspA-GFP protein, or the inactive UspA^AA^-GFP mutant or mere GFP as a negative control. The cultures were inoculated with 10^6^ spores/mL in minimal medium and were grown for 20 h vegetatively at 37 °C in the presence of light. Elution fractions were analyzed with LC-MS, and putative interaction partners of UspA-GFP, UspA^AA^-GFP are shown in the heat map. The heat map represents the log2 of the label-free quantification (LFQ) values of the co-purified proteins. Identified proteins were assigned into different categories that are represented in pie charts.

GFP pull-down experiments were performed to identify potential interaction partners of UspA-GFP and the inactive UspA^AA^-GFP mutant. The mutated UspA^AA^-GFP version is presumably less dynamic than the active fusion protein. This might lead to the identification of proteins, which interact only shortly with active UspA. Strains were grown under submerged agitated culture conditions, and whole cell lysates were used for GFP pull–downs. Elution fractions were subjected to trypsin digestion and subsequent analysis by LC-MS. Both protein versions pull 38 common proteins, 15 proteins that were identified only in pull-downs of UspA^AA^-GFP and seven proteins that were exclusively identified in the pull-down of the functional UspA-GFP fusion protein (Figure 3, Table S5).

Among the 38 commonly identified proteins, the GFP-coupled bait UspA was identified in all replicates. Approximately one-third of these proteins are associated with primary metabolism. Five proteins related to nuclear transport, including the two karyopherins KapB and KapF, were captured by both protein versions. KapB and KapF are essential proteins for *A. nidulans* development [78]. This corroborates that UspA exerts functions at the nucleo/cytoplasmic interface. Four proteins related to fungal development, e.g., the cell wall mannoprotein MnpA and the Guanyl-nucleotide exchange factor (HypB), were identified. HypB is involved in hyphal growth of *A. nidulans* [79]. UspA associates to UspF, another putative DUB of the USP family. The transcription of the *uspF* gene expression is upregulated in the deletion strain of the CSN subunit CsnE as well (Table S5, Figure 1). The interaction between CSN and UspA in undifferentiated, vegetative hyphae is presumably not tight, because only some CSN subunits were identified with single peptides in some biological replicates below the threshold set for data analysis for putative interaction partners of UspA. This includes PCI domain containing subunits CsnA, CsnD and CsnG as putative interaction partners of active as well as inactive UspA.

The inactive UspA^AA^-GFP fusion protein pulled exclusively 15 proteins that were not identified in the pull-downs of the active UspA-GFP, including the Ser/Thr kinase PhoB or the beta-1,4-endoglucanase EglD. The inactive UspA^AA^-GFP fusion protein could be less dynamic and is able to capture polyubiquitin, which hints to the possibility that UspA recognition and binding to an ubiquitin chain might be more important than the binding to the ubiquitinated protein itself.

### 3.5. UspA Reduces the Cellular Pool of Ubiquitinated Proteins in A. nidulans

**Figure 4:**
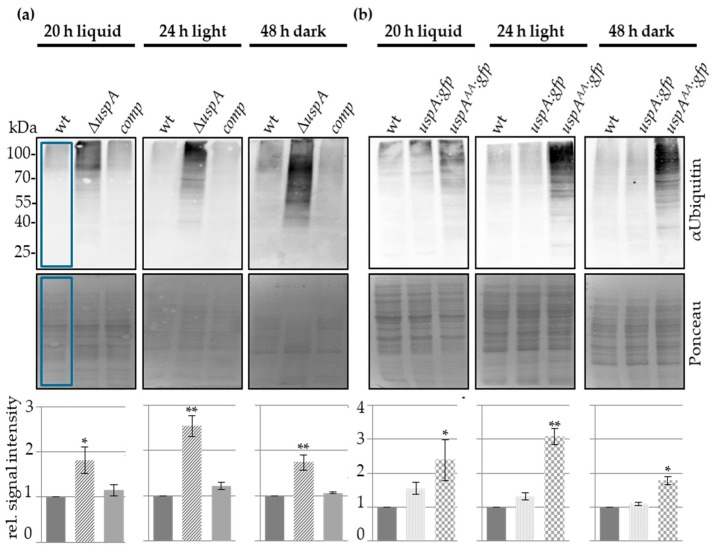
UspA balances the amount of total ubiquitinated proteins during fungal development. Western blot experiments were performed with protein crude extracts of wild type (wt), Δ*uspA*, Δ*uspA* complementation (comp) (**a**), as well as of *A. nidulans* strains expressing the functional *uspA:gfp* fusion or the inactive *uspA^AA^:gfp* fusion (**b**). All strains were grown for 20 h in minimal liquid medium at 37 °C to enable vegetative growth. The mycelium derived from this vegetative development was transferred onto solid agar plates that contained equal amounts of minimal medium and incubated either for 24 h in light at 37 °C to induce asexual development or for 48 h in darkness at 37 °C to induce sexual development. As a loading control, the proteins on the nitrocellulose membrane were stained with Ponceau. The whole lane, marked with a blue frame exemplarily for wild type, was used for the quantification of the signal intensity and normalized to the Ponceau signal with the Bio-1D software. The quantification is based on up to four biological replicates. The standard error of the mean is shown, and significances of differences between wt and Δ*uspA* or between wt and *uspA^AA^:gfp* were calculated with *t*-test (** *p* ≤ 0.01; * *p* ≤ 0.05).

For further characterization of UspA, we were interested in the effect of UspA on the ubiquitination pattern of proteins in *A. nidulans*. Therefore, different strains defective in the function of the ubiquitin-specific protease UspA were used to analyze the effect of UspA on the ubiquitination of proteins. An *uspA* deletion strain was constructed by replacing the *uspA* ORF by the *pyroA* marker cassette derived from *A. fumigatus*. Western blot experiments with total cellular crude extracts from different developmental time points revealed that the cellular amount of total ubiquitinated proteins increased significantly in the absence of *uspA* compared to wild type during all tested growth conditions. Ectopic integration of the *uspA* ORF into the deletion strain complemented the ubiquitination phenotype (Figure 4a). The expression of the *uspA:gfp* fusion is functional and did not change the number of total ubiquitinated proteins compared to wild type. The *uspA^AA^:gfp* mutation abolished the deubiquitination activity, resulting in similar increased amounts of ubiquitinated proteins as the *uspA* deletion strain. This indicates that the two conserved cysteine residues, C469 and C1066, are essential for the enzymatic activity of UspA. UspA represents a major ubiquitin-specific protease, which is active throughout the whole fungal life cycle, where it deubiquitinates a broad range of proteins (Figure 4b). The comparably high number of ubiquitinated proteins in *uspA* deficient strains, together with the identification of polyubiquitin in GFP pull-downs of the UspA^AA^-GFP expressing strain, suggests further that UspA may recognize directly ubiquitin chains instead of specific substrates, which, in turn, points to a broad substrate spectrum of the deubiquitinase UspA.

### 3.6. UspA Accelerates the Formation of Asexual Conidiospores and Sexual Fruiting Bodies in A. nidulans

**Figure 5:**
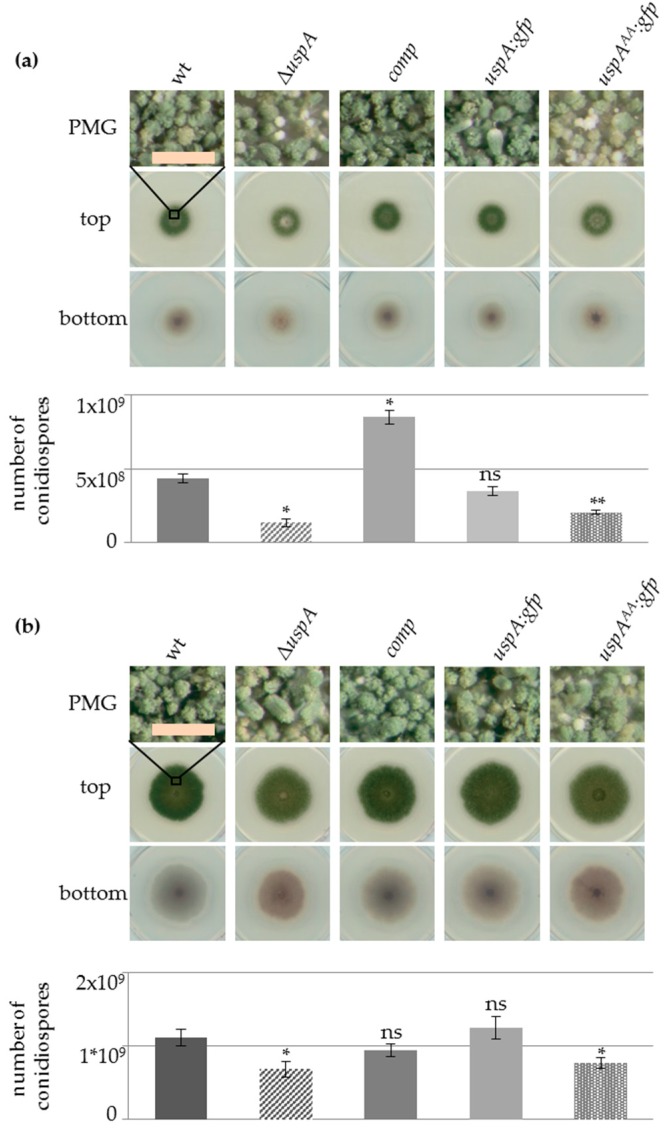
UspA accelerates the formation of conidiospores. 5000 spores were point-inoculated on minimal medium and incubated for three days (**a**) or five days (**b**) at 37 °C in light. A photomicrograph (PMG) of the highlighted region (black square), as well as the top and bottom of the colonies, are shown (comp: ectopic expression of *uspA* in Δ*uspA*). The error represents the standard error of the mean of three biological replicates with two technical replicates each. Significances against wild type (wt) number of spores were calculated with *t*-test (** *p* ≤ 0.01, * *p* ≤ 0.05, ns – not significant). The scale bar in the PMG represents 200 μm.

The accumulation of total ubiquitinated proteins especially during asexual development (Figure 4), as well as the increased transcript levels of *uspA* in the absence of CsnE (Figure 1), suggests that UspA has an impact on fungal development and secondary metabolism. Conidiospore formation of the *uspA* deletion strain, the respective complementation, the *uspA:gfp* fusion protein expressing strain, as well as the strain expressing the inactive *uspA^AA^:gfp* mutant were analyzed after three and five days of development in light. The colonies of Δ*uspA* and the *uspA^AA^:gfp* expressing strain were characterized by a lighter green color, indicating already the formation of fewer conidiospores and a change in the pigmentation at the bottom of the colony, which indicates altered secondary metabolism. The deletion strain formed 30% of the amount of wild type conidiospores after three days of development, whereas the strain carrying the inactive mutant produced 46% of wild type conidiospores. The strain expressing the functional *uspA:gfp* fusion protein showed no significant differences in conidiospore formation in comparison to the wild type. The strain with ectopic complementation of Δ*uspA* (*comp*) formed an increased number of conidiospores after three days of development, but, after five days, it showed no significant difference to the wild type anymore. Strains deficient in UspA function show still significantly fewer conidiospores after five days compared to the strains carrying a gene for the functional deubiquitinase (Figure 5). This suggests that the catalytic activity of UspA is essential for the timely coordination of conidiospore formation.

**Figure 6:**
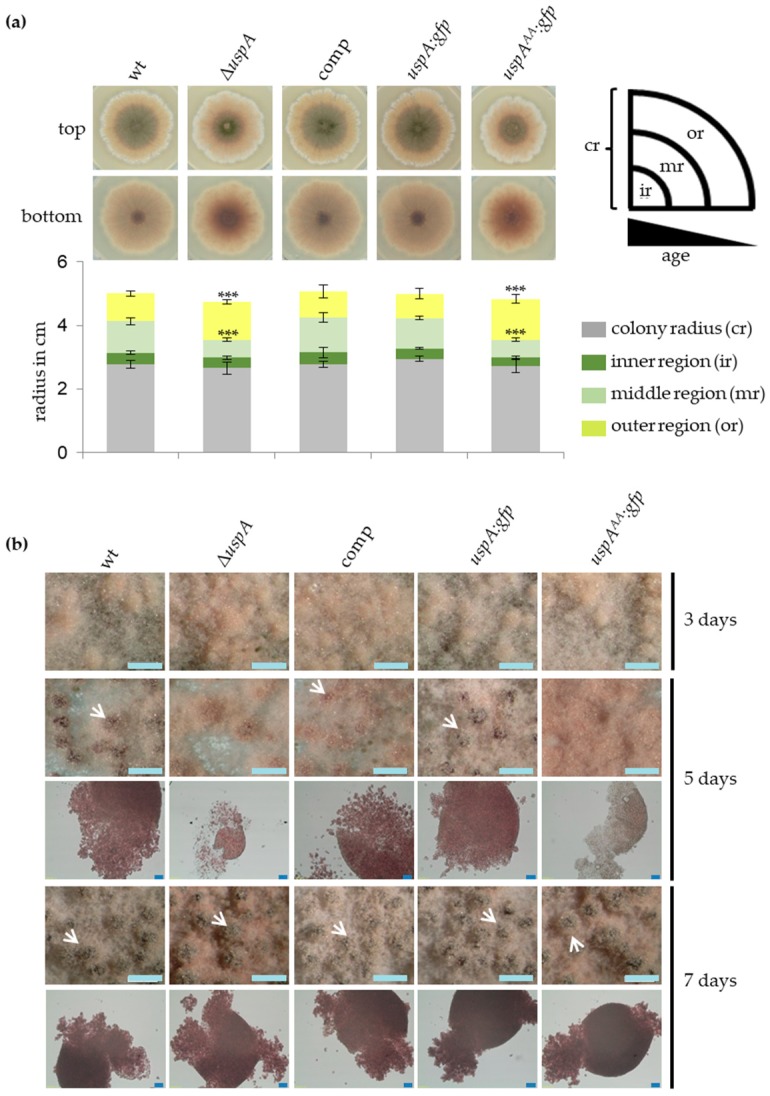
UspA is necessary for the early steps of cleistothecia development. (**a**) Point-inoculation of 5000 spores of the indicated strains on solid agar plates containing minimal medium was performed. Plates were incubated for seven days at 37 °C in darkness. The radius of different colony regions was measured. Error bars represent the standard deviation of at least three biological replicates, significances were calculated with t-test (*** *p* ≤ 0.001). (**b**) For each strain, 30000 spores were equally distributed on an agar plate containing a minimal medium. Plates were incubated for three, five, or seven days at 37 °C in darkness. The white arrows indicate cleistothecia. The light blue scale bar represents 200 µm, dark blue scale bars represent 50 µm.

In darkness and under oxygen-limiting conditions, *A. nidulans* favors the formation of cleistothecia as sexual fruiting bodies, which serve as overwintering structures. The formation of cleistothecia was investigated in strains defective in UspA function. Similar as in asexual development, the expression level of *uspA* was increased in the Δ*csnE* strain during sexual growth conditions (Figure 1), and the total ubiquitination level of proteins was significantly increased in strains defective in UspA function (Figure 4). *A. nidulans* strains without CsnE or any other CSN subunit arrest at the level of primordia and are unable to form mature cleistothecia [26]. *A. nidulans* strains form different circular regions when point-inoculated and incubated under sexual development inducing conditions. Point-inoculation of *A. nidulans* strains allows the analyses of different maturation steps at the same time, as the middle of the colony is the oldest (most mature), and the outermost region of the colony consists of very young mycelium. The middle region (mr) that contains mature cleistothecia was reduced in *uspA* deficient strains, whereas the outer region (or) containing immature fruiting bodies was enlarged in *uspA* deficient strains compared to the control strains. Since no difference in the total colony radius (cr) was observed, *uspA* deficient strains have a developmental but not a general growth defect. After three days of development, the *uspA* deficient strains formed early nests and immature fruiting bodies that were not fully pigmented. In addition to the early nests, wild type, complementation (comp), and *uspA:gfp* strains developed dark-colored fruiting bodies that contain many ascospores. After seven days, mature cleistothecia were formed in all strains. Thus, UspA is not essential for the formation of cleistothecia as the CSN complex but required to accelerate the early steps of fruiting body formation (Figure 6).

### 3.7. UspA Represses the Expression of Derivative of Benzaldehyde (dba) Gene Cluster Members

**Figure 7:**
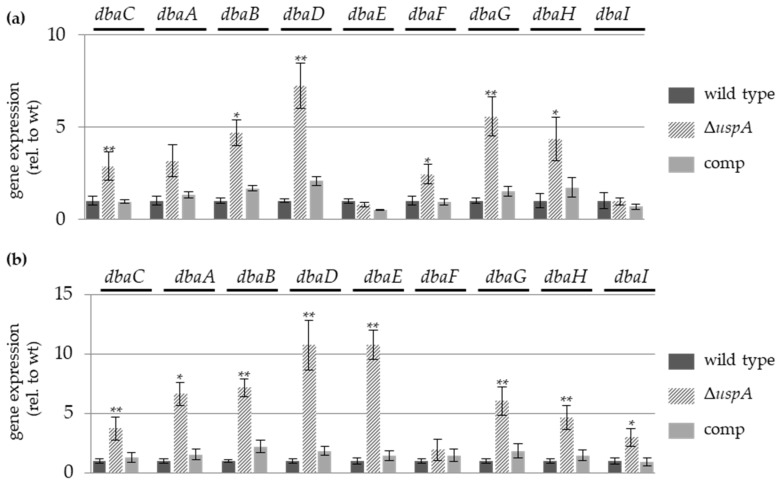
UspA represses the expression of the *dba* gene cluster. qRT-PCRs were performed for *A. nidulans* wild type, Δ*uspA,* and the ectopic complementation (comp) strain. Mycelium of respective strains was transferred after 20 h of growth in submerged culture onto solid agar plates and incubated for 24 h in light (**a**) to induce asexual development or (**b**) 48 h in darkness to induce sexual development. Gene expression was normalized against *h2A* and *15S rRNA.* Error bars represent the standard error of the mean of three biological and three technical replicates, each (* *p* ≤ 0.05; ** *p* ≤ 0.01).

During asexual as well as sexual development of the *uspA* deletion strain, a change in the color of the bottom of the colony occurred, which indicates altered secondary metabolism (Figure 5 and Figure 6). The *derivative of benzaldehyde* (*dba*) gene cluster is upregulated in *csnE* deletion strains, which is deficient in deneddylation activity [80]. The product of this gene cluster is 2,4-dihydroxy- 3-methyl-6-(2-oxopropyl) benzaldehyde (DHMBA) and provides antibacterial activity against gram-positive bacteria [80]. Due to the UspA interaction with several subunits of the COP9 signalosome, it was analyzed whether the *uspA* deletion strain showed increased gene expression of the *dba* gene cluster as *csn* mutant strains. Genes of the *dba* cluster were upregulated in Δ*uspA* compared to wild type and complementation strain during both multicellular developmental pathways. The highest upregulation was observed for the substrate transporter encoding gene *dbaD*, which was up to eight times higher in the deletion strain compared to the wild type. *dbaG*, which is a putative transcription factor, also showed increased expression levels in the absence of UspA during asexual and sexual development conditions. This supports that UspA and CsnE are required for the repression of the *dba* gene cluster and that deubiquitinase and deneddylase cooperate in controlling the coordination of *A. nidulans* multicellular development and secondary metabolism (Figure 7).

### 3.8. UspA Prevents Accumulation of the Velvet Domain Protein VeA During Late Asexual and Sexual Development

**Figure 8:**
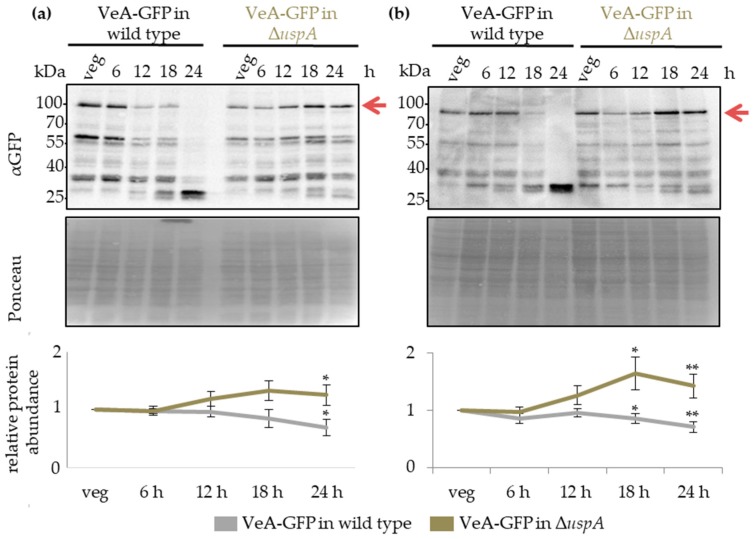
UspA prevents the accumulation of VeA-GFP during multicellular development. Strains were grown in submerged culture for 20 h at 37 °C (veg) and shifted onto solid agar plates containing equal amounts of minimal media. Plates were incubated for 24 h at 37 °C in light and in darkness to induce asexual (**a**) or sexual (**b**) development, respectively. Samples were taken every six hours. Protein crude extracts were prepared to analyze the abundance of VeA-GFP fusion protein during developmental stages in wild type or Δ*uspA* background strains through western blot experiments. The quantification of the VeA-GFP fusion protein abundance is based on five biological with two technical replicates each. Error bars represent the standard error of the mean). Significances were calculated with t-test and always normalized to the Ponceau staining and the fusion protein abundance during vegetative growth (* *p* ≤ 0.05; ** *p* ≤ 0.01).

The velvet domain protein VeA is a key regulator of fungal development and secondary metabolism. It is localized in the cytoplasm and shuttles into the nucleus in response to external or internal stimuli. VeA nuclear entry and stability are reduced during asexual development by proteins as the methyltransferases VipC-VapB [41,81]. The velvet complex formed by VeA together with VelB and the methyltransferase LaeA, is only formed in the nucleus [37]. A VeA-GFP fusion protein was expressed under the native *veA*-promoter, and protein abundance during fungal development was monitored in wild type compared to Δ*uspA* strain. The amount of VeA-GFP fusion protein decreased in the wild type during the first 24 h of development but increased in Δ*uspA* by approximately two-fold after 24 h of asexual or sexual differentiation (Figure 8).

The VeA-GFP accumulation in *uspA* deletion strains could cause the delay in conidiospore and cleistothecia formation in the mutant compared to the wild type strain. The altered protein abundance of VeA in the absence of UspA did not interfere with the subcellular localization of VeA or its ability to interact with VelB (Figure S3). The data suggest that VeA has an important role when the fungus reaches developmental competence and at the promotion of initial steps of multicellular development.

### 3.9. Fbx23 Prevents Accumulation of VeA-GFP in Late Developmental Time Points

Deubiquitinating enzymes stabilize their substrates due to deubiquitination and can prevent their degradation by the 26S proteasome [82]. UspA dependent destabilization of VeA-GFP during ongoing later stages of development could, therefore, be an indirect effect. The mammalian β-TrCP Fbx protein targets IκBα for ubiquitination and degradation. IκBα acts as an inhibitor of NF-κB transcription factor nuclear entry, which has the same fold in the DNA binding and dimerization domain as the velvet domain protein VeA [38,83]. BLAST analyses revealed that Fbx23 is the putative ortholog of the mammalian β-TrCP Fbx protein, which binds IκBα and initiates its destruction. *A. nidulans* carries genes for more than 70 different Fbox proteins in its genome. Fbox proteins are required for the specific binding of substrates for subsequent 26S proteasome-dependent degradation [84]. Fbx23-SCF complexes accumulate in cells defective in CSN in *A. nidulans,* and the gene for this Fbx protein is essential for light-dependent asexual development as well as alternative carbon source utilization [84,85]. Numerous Fbx23 interacting proteins have been identified, but they did not include VeA [85]. Interaction between UspA and Fbx23 might be sensitive to protein tags, because GFP-tagged Fbx23 fusion protein prepared to monitor Fbx23 protein abundance did not rescue the wild type phenotype, regardless of whether a recyclable selection marker was used. In accordance with the mammalian system, Fbx23 could bind VeA interacting and destabilizing proteins, and loss of Fbx23 might stabilize VeA. This was analyzed by comparing VeA-GFP levels in wild type and Δ*fbx23* background strains.

**Figure 9:**
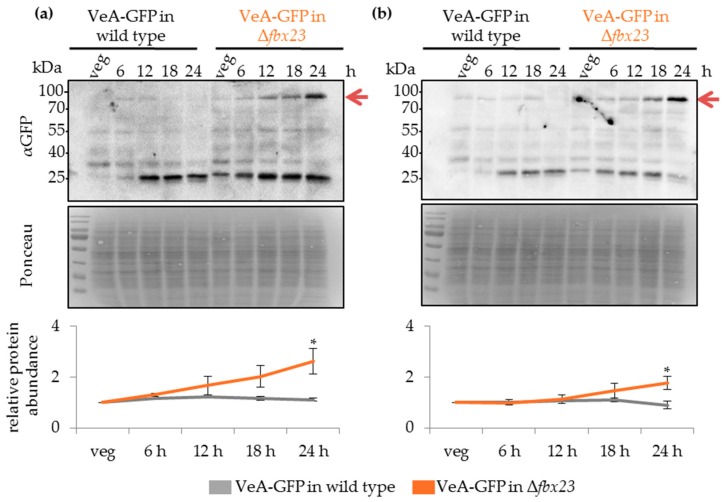
Fbx23 prevents VeA-GFP accumulation during the late stages of multicellular development. Strains were grown in submerged culture for 20 h at 37 °C (veg) and shifted onto solid agar plates containing equal amounts of minimal media. Plates were incubated for 24 h at 37 °C in light or in darkness to induce asexual (**a**) or sexual (**b**) development, respectively. Samples were taken every six hours. Protein crude extracts were prepared to analyze the abundance of VeA-GFP fusion protein during developmental stages in wild type or Δ*fbx23* strain. Signals of VeA-GFP fusion protein were normalized against Ponceau staining of the nitrocellulose membrane. Quantification results of four biological replicates are shown. The standard error of the mean is shown. Significances were calculated with the *t*-test, always compared to the protein amount during vegetative growth (* *p* ≤ 0.05).

Western blot experiments with total protein crude extracts derived from vegetatively grown cultures, as well as from different time points, after the initiation of asexual or sexual development, were performed. VeA-GFP fusion protein accumulated during asexual and sexual development up to 24 h upon the deletion of *fbx23* compared to wild type (Figure 9). The impact of VeA-GFP protein accumulation during multicellular development was similar when deubiquitinating enzyme UspA or the Fbx23 was missing. Both proteins prevented an accumulation of VeA-GFP during late developmental time points. UspA could either directly target another substrate of Fbx23, which influences VeA abundance, or UspA could deubiquitinate and stabilize Fbx23 directly, which hinders Fbx23 at the ubiquitination and destabilization of a so far unknown protein that, in turn, influences VeA protein abundance.

## 4. Discussion

**Figure 10:**
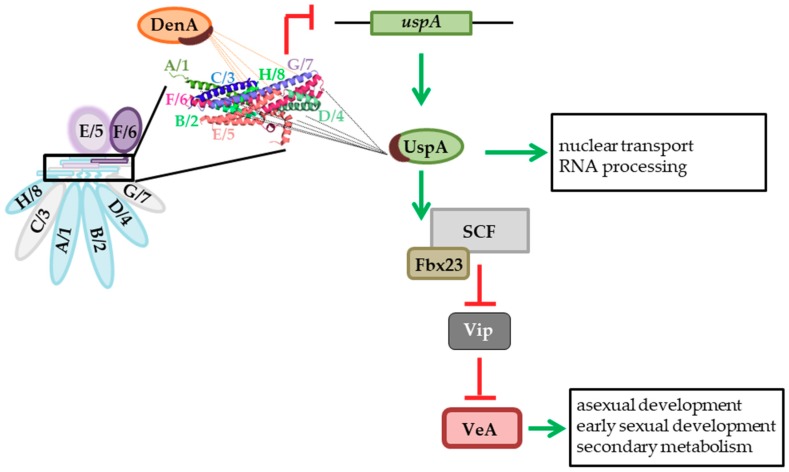
UspA interacts with the COP9 signalosome and influences the abundance of VeA. The deubiquitinase UspA interacts with six subunits of the COP9 signalosome (shown schematically in blue (PCI domain subunits) and in purple (MPN domain subunits)). The interaction surface of UspA might be at the helical bundle, where the C-terminal helices of all CSN subunits are connected. The crystal structure of the human COP9 signalosome was resolved in 2014 [22]. The model provided in the protein data bank (PDB 4d10) was edited with PyMOL 2.0 software and the C-terminal helices, which form the helical bundle, are shown here. The interaction surfaces of DenA and UspA with the COP9 signalosome are not mutually exclusive. The presence of an intact COP9 signalosome represses the gene expression of *uspA*. UspA co-purifies proteins related to nuclear transport or transcriptional processing. The presence of UspA and the presence of the Fbx23 leads to degradation of the key regulator of fungal development and secondary metabolism, VeA, after the initiation of multicellular development. A defect of UspA leads, therefore, to the delayed formation of developmental structures and to altered secondary metabolism. Whether VeA is targeted for ubiquitination by Fbx23 directly or if Fbx23 binds and, in turn, leads to ubiquitination of a VeA-interacting protein (Vip) remains to be addressed in future studies.

We demonstrate here that the deubiquitinating enzyme UspA/15 and the Fbx protein 23/β-TrCP are involved in similar regulatory mechanisms to control fungal regulation of multicellular development and secondary metabolism mediated by the velvet domain protein VeA and mammalian infection response mediated by the Rel-homology domain protein NF-κB. A summary of the findings of this study is shown in Figure 10.

The COP9 signalosome and DenA/1 are two interacting and conserved deneddylases that regulate the activity of the ubiquitin-proteasome system (UPS) [24,26,35,72]. The eight-subunit COP9 signalosome serves as an interaction platform for several proteins that regulate protein half-life, among them, are different kinases and the second deneddylase DenA/1 [35,72,86,87]. CSN co-purifies in mammalian cell lines with Den1, whereby the interaction could be mapped by far western blots to CsnA/1 and CsnB/2 [35]. Direct protein-protein interactions of the ortholog in *A. nidulans*, DenA/1, were supported by Y2H experiments for CsnA/1, E/5, F/6, and G/7, whereby the strongest interaction was observed with the PCI domain-containing subunit CsnG/7 [35]. Furthermore, CsnC/3, E/5, F/6, G/7, and H/8 have a destabilizing effect on DenA/1 [72].

Here we showed that *A. nidulans* UspA, a deubiquitinating enzyme, is another interaction partner of the COP9 signalosome. It can interact directly with CsnA/1, B/2, D/4, E/5, F/6, and CsnH/8 and is, therefore, another regulatory factor that is recruited through CSN to the ubiquitination machinery. The orthologous proteins in human and *S. pombe*, Usp15 and Ubp12, were co-purified with CSN, but CSN subunits responsible for this interaction remained elusive [19,20]. UspA is not required for the vegetative growth of *A. nidulans* under normal conditions, and accordingly CSN subunits as potential UspA interaction partner were only identified in GFP pull-down experiments from undifferentiated hyphae below the threshold set for mass spectrometry analyses. UspA is important for multicellular development and might be required under certain stress conditions when the UspA-CSN interaction might be more important. The large UspA subpopulation, which is present in undifferentiated *A. nidulans* hyphae and which is not interacting with CSN subunits, suggests that there is also a function of UspA independently of CSN. The interaction of the second deneddylase DenA/1 and the deubiquitinase UspA/15 with the COP9 signalosome are presumably not mutually exclusive as the main interacting subunits are different. Currently, it is yet unknown whether the interaction takes place at the same time or during different developmental stages or environmental conditions. The interaction of DenA with the COP9 signalosome is located mainly inside nuclei, whereas a subpopulation of DenA is present in the cytoplasm as well [35,72]. In contrast, the interaction of UspA with CSN subunits is predominantly localized to the nuclear periphery, with a smaller subpopulation inside the nucleus. Even though a simultaneous binding of UspA and DenA to the CSN would be sterically possible, they might be restricted to certain cellular compartments.

The coordination of *A. nidulans* multicellular development and secondary metabolism is strongly related to changes in gene expression, which is propagated to significant changes in the fungal proteome generated by the interplay between protein synthesis and degradation [88]. DenA supports the formation of asexual conidiospores and the repression of fruiting body formation in light. The DenA-CSN interaction inside the nucleus and DenA functions at septae might regulate proteins required for these developmental processes [72]. In comparison, UspA is required at the initiation of asexual and sexual development but not for the formation of conidiospores or cleistothecia. As it is located at the nuclear periphery, it might be involved in the nuclear/cytoplasmic transport of proteins that initiate these developmental processes.

The COP9 signalosome coordinates through its complex interaction partners multicellular development of *A. nidulans*. Only the fully assembled eight-subunit COP9 signalosome can deneddylate CRLs, and the catalytically active subunit CsnE/5 is the last subunit incorporated into the stable seven-subunit pre-CSN and mainly localized inside nuclei [22,26,32,74,75,76]. Several additional CSN subcomplexes consisting of two up to four subunits have been described in different organisms like *S. pombe*, *Arabidopsis thaliana,* or mammalian cell lines, which can be localized in the cytoplasm or in the nucleus [73,74,75,76]. The function and the composition of the subcomplexes are not well understood. UspA could be involved in the cytoplasmic/nuclear shuttling of CSN subunits or subcomplexes from the cytoplasm to the nucleus. It is yet unknown whether the location of CSN or CSN subcomplexes differs in the presence or absence of UspA. The association of UspA with several nuclear transport factors, like the two essential karyopherins KapB and KapF, and its localization close to and a small population even inside nuclei point to a role of UspA in nuclear transport. How this mechanically works, and if UspA might remove ubiquitin molecules from proteins to allow or prevent their nuclear entry or if UspA stabilizes proteins of the nuclear transport machinery through deubiquitination to allow nuclear transport, remains to be clarified.

Inside the nucleus, the trimeric velvet complex, consisting of the two velvet domain containing proteins VeA and VelB and the methyltransferase LaeA, induce sexual development and the concomitant secondary metabolism [89]. VeA is located in the cytoplasm during light conditions. Dependent on certain environmental stimuli or the absence of light, VeA travels together with VelB into the nucleus to fulfill its function [89,90]. VeA is required when *A. nidulans* reaches developmental competence and is able to react on external and internal stimuli. After the decision and initiation of the corresponding developmental program, VeA is not required anymore and becomes degraded. In *uspA* deficient strains, however, VeA is not degraded but accumulates in the cell. This change in protein abundance of this key developmental regulator could be the reason for an imbalance in the formation of VeA-containing protein complexes inside and outside of the nucleus and thereby causing the observed delay in multicellular development in *uspA* deletion strains. The accurate control of VeA-containing homo- and heterodimers is important for the correct tuning of fungal development. VeA competes with the velvet domain protein VosA for interaction with VelB. VeA-VelB supports sexual development and the appropriate secondary metabolism, whereas VosA-VelB reduces asexual development, including large regulatory networks with overlapping and opposite subnetwork functions [37,48]. *A. nidulans* UspA could deubiquitinate a broad spectrum of proteins, including velvet domain regulators, during the entire fungal development, when it is located at the interface of nuclear periphery and nucleus. The identity of the specific UspA substrates remains still elusive. As deubiquitination reactions are fast and the interaction between the DUB and its substrate or other proteins might be not very strong, the inactive UspA^AA^-GFP might interact longer with respective interaction partners. The identification of polyubiquitin only in the elution fraction of the inactive fusion protein suggests that UspA rather recognizes and binds the ubiquitin chain than the labeled protein itself. 

This limited substrate specificity allows the deubiquitination and protection of various SCF complexes that are bound to different substrate receptor proteins during their nuclear/cytoplasmic shuttling. Fbox proteins can be protected while they are searching for substrates in the cell. UspA might stabilize, through its deubiquitination activity, not only Fbox proteins but also other CRL components or CSN subunits.

Degradation of proteins through the UPS requires Fbox protein substrate receptors, which bind the substrate prior to ubiquitination. In *csnE* deficient *A. nidulans* strains, SCF complexes bound to Fbx proteins 1, 2, 15, and 23 accumulate [84]. Absence of Fbx23 leads, similarly to *uspA* deletion, to an accumulation of VeA-GFP during late development. This leads to the conclusion that Fbx23 might target VeA for ubiquitination and, in turn, UspA protects Fbx23 for autoubiquitination of the SCF complex. It is yet unclear, whether Fbx23 is a direct target of UspA, which would be similar to human cells. Mammalian β-TrCP Fbx protein is closely related to *A. nidulans* Fbx23, and the RHD domain of NF-κB and the velvet domain of VeA share a similar fold for DNA binding and dimerization. β-TrCP targets the physically interacting inhibitor of NF-κB for ubiquitination and subsequent degradation [38,91,92]. The Fbx23-mediated degradation of a velvet interacting protein or rather a protein that controls VeA subcellular localization could be an explanation for the observed VeA accumulation. Besides LaeA, two other methyltransferases, namely VipC-VapB, can interact with VeA. Both proteins counteract the nuclear import of VeA, and VipC decreases VeA stability [41]. By ubiquitination and subsequent degradation of VipC through Fbx23, the abundance of VeA can be increased. UspA might counteract VipC ubiquitination. In the absence of a functional UspA, VipC cannot be protected anymore, which leads to degradation and a concomitant increase in VeA abundance. Like in the mammalian system, the controlled ubiquitination and degradation or deubiquitination and protection of proteins that coordinate nuclear import of transcription factors or regulatory proteins is essential for a functional immune response or, here, coordinated fungal development. The phosphorylation and subsequent ubiquitination and degradation of IκBs allow the translocation of the mammalian NF-κBs into the nucleus, whereby deubiquitination mediated by Usp15, which is bound to the COP9 signalosome, counteracts this process [91]. In, *A. nidulans*, VipC could be the equivalent to IκBs and a putative target of UspA, which needs to be addressed in future studies.

This study shows the importance of deubiquitinases in coordinating and accelerating single steps of fungal development and controlling secondary metabolism in the ascomycete *A. nidulans* and paves the way for future investigations on deubiquitinating enzymes in fungi. Deregulation of the proteome homeostasis will provide useful insights into so far uncharacterized secondary metabolite gene clusters. Due to the high conservation of the ubiquitin system from fungi to humans, new findings will provide the basis for further medical or industrial research and also reveal novel aspects in the evolution of both organisms after the separation from a common ancestor about one billion years ago.

## 5. Conclusions

UspA of the mold *Aspergillus nidulans* corresponds to human Usp15 and is one representative of a large repertoire of largely unstudied deubiquitinating enzymes in filamentous fungi. UspA interacts in the periphery and inside of the nucleus with import and export proteins and with the COP9 signalosome. UspA controls, together with the substrate receptor Fbx23 of E3 cullin-RING ubiquitin ligases, the cellular amount of the velvet domain protein VeA as a central initial regulator of fungal development and concomitant secondary metabolism. The velvet domain folds as the structurally similar RHD domain of mammalian NF-κB transcription factors, which indicates a common origin between fungal development and secondary metabolism and the immune response pathway in mammalian cells as survival and defense mechanisms. A large number of yet unexplored fungal deubiquitinating enzymes is a promising reservoir for the identification of novel bioactive metabolites for potential pharmaceutical or agricultural applications.

## Figures and Tables

**Table 1:** The repertoire of deubiquitinating enzymes in *A. nidulans*.

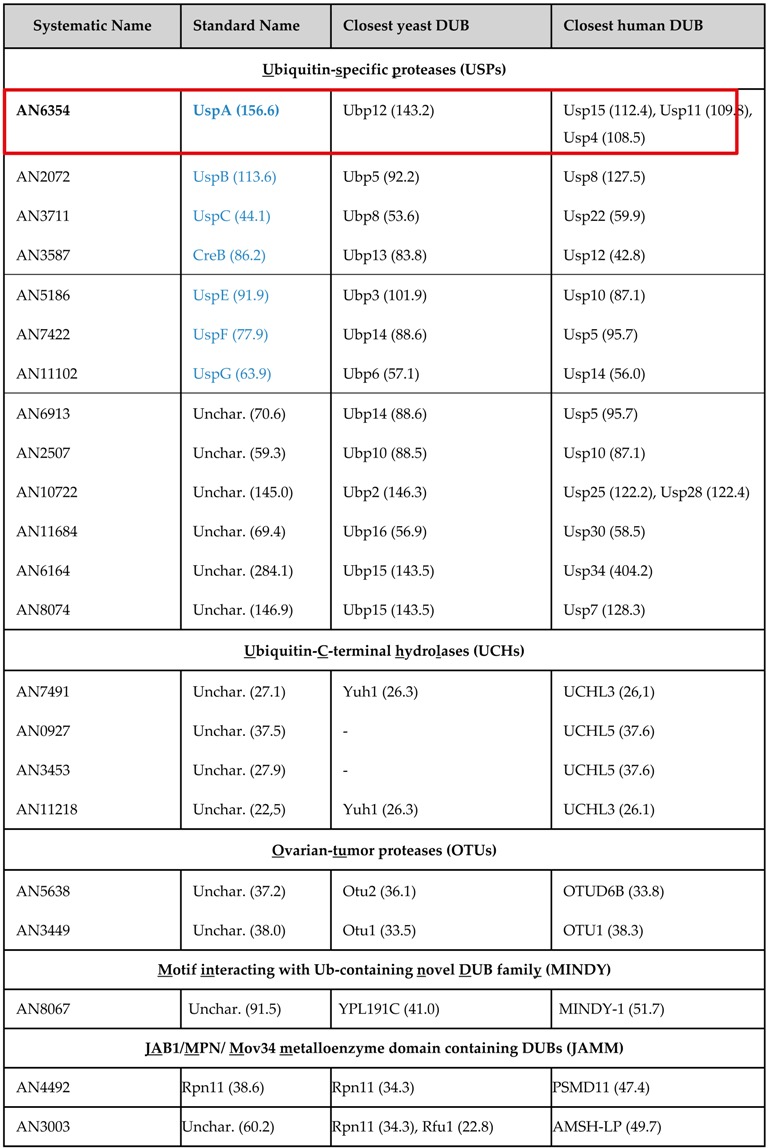

A text based search of proteins with predicted ubiquitin hydrolase activity was performed in AspGD [56] and FungiDB [57]. BLAST analyses [58] were performed with NCBI Blastp of protein sequences of *A. nidulans* against the RefSeq database of *Saccharomyces cerevisiae* (yeast) (TaxID: 4932) or *Homo sapiens* (human) (TaxID: 9606). Related proteins in yeast and human with the lowest E-value are shown. In brackets, the molecular weight of the proteins in kDa according to UniProt is given. The genes encoding the blue marked proteins were used in qRT experiments in this study. The red box emphasizes UspA. DUB: deubiquitinating enzyme.

## Supplementary Materials

The following are available online at https://www.mdpi.com/2218-273X/9/6/238/s1. **Figure S1:** *A. nidulans* UspA has approximately 30% sequence identity to human Usp15, Usp4, and Usp11. **Figure S2:** The amount of ubiquitinated proteins is not affected by CsnE. **Figure S3:** Localization of VeA and its interaction with VelB are independent of UspA. **Table S1:** Plasmids used in this study. **Table S2:** *A. nidulans* and *S. cerevisiae* strains used in this study. **Table S3:** Oligonucleotides used for plasmid construction in this study. **Table S4:** Oligonucleotides used for qRT experiments. **Table S5:** Functional groups of proteins identified in GFP pull-downs of UspA-GFP and UspA^AA^-GFP.
