# Supplementary material for: COP9 Signalosome Interaction with UspA/Usp15 Deubiquitinase Controls VeA-Mediated Fungal Multicellular Development
Source: Biomolecules. 2019 Jun 18;9(6):238. doi: 10.3390/biom9060238 (PMC6627422; doi:10.3390/biom9060238)
Supplement: Supplementary file 1 [file biomolecules-09-00238-s001.pdf]

Supplementary Figures and Tables

COP9 signalosome Interaction with UspA/Usp15 Deubiquitinase Controls VeA-Mediated Fungal Multicellular Development

Cindy Meister[1], Karl G. Thieme[1], Sabine Thieme[1], Anna M. Köhler[1], Kerstin Schmitt [1], Oliver Valerius[1] and Gerhard H. Braus[1*]

[1]Department of Molecular Microbiology and Genetics and Goettingen Center for Molecular Biosciences (GZMB), University of Goettingen, 37077 Goettingen, Germany; ; cmeiste@gwdg.de (C.M.); kthieme@gwdg.de (K.G.T.); sreen@gwdg.de (S.T.); akoehle3@gwdg.de (A.M.K.); kschmit1@gwdg.de (K.S.); ovaleri@gwdg.de (O.V.)

*Correspondence: Gerhard H. Braus, gbraus@gwdg.de; Tel.: +49-551-39-33771

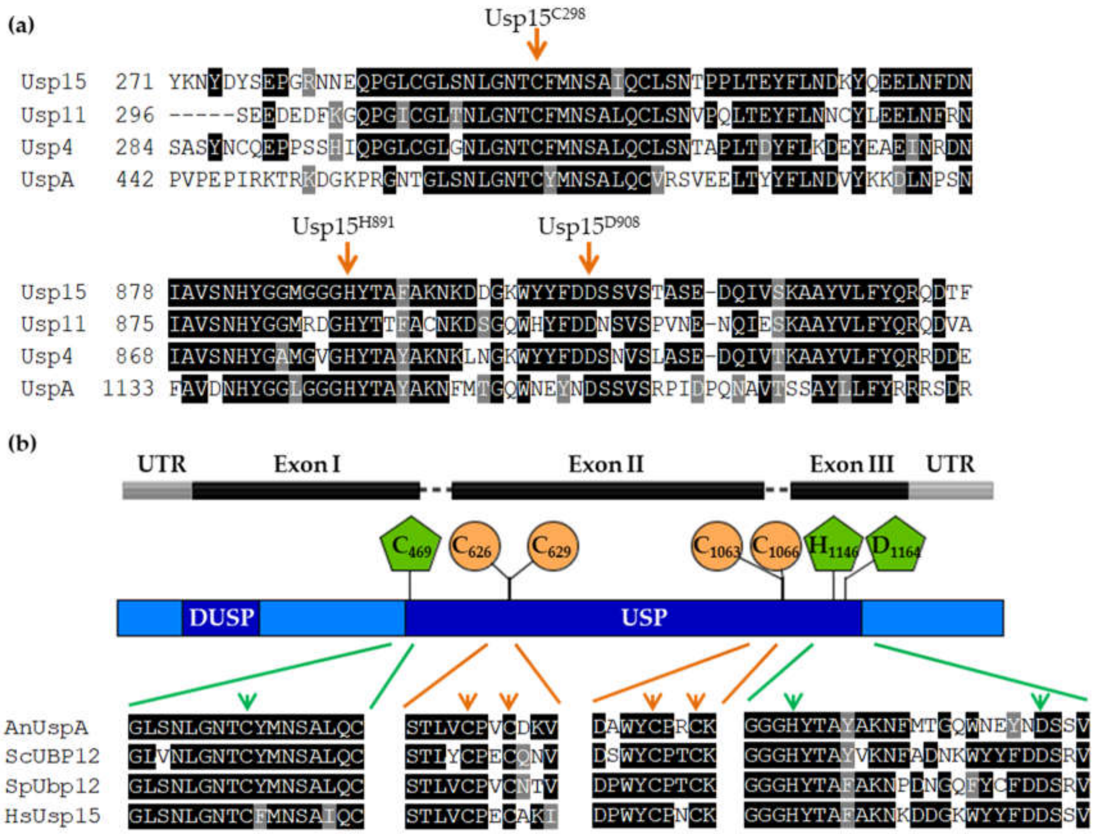

(c)

Protein	Length in amino acids	Molecular weight (kDa)
AnUspA	1418	156.6
HsUsp15	981	112.4
HsUsp11	963	109.8
HsUsp4	963	108.6
ScUBP12	1254	143.2
SpUbp12	979	111.9

Figure S1. *A. nidulans* UspA has approximately 30 % sequence identity to human Usp15, Usp4 and Usp11. **(a)** Alignment of protein sequences of human Usp15 (Q9Y4E8), Usp11 (P51784) and Usp4 (Q13107) with the fungal AN6354 (UspA, Q5AZC6) was performed using Clustal Omega alignment tool. Residues were coloured due to their similarity using the Boxshade online tool (version 3.21). The catalytic triad of Usp15 consists of C298, H891 and D908 [93]. These residues are conserved in human Usp11 and Usp4, as well as in the putative *A. nidulans* ortholog UspA. **(b)** UspA is encoded by a gene encompassing 4348bp, three exons and two introns. The protein is characterized by an N-terminal DUSP (domain specific for ubiquitin-specific proteases) domain and a 729 amino acid encompassing catalytic domain that contains the residues of the catalytic triad (in green) and zinc finger motifs (in orange) that are highly conserved in fungi like *S. cerevisiae* (P39538) or *S. pombe* (O60079) and human. **(c)** An overview about the length of the homologous proteins and their molecular weight is given according to information given on UniProt.

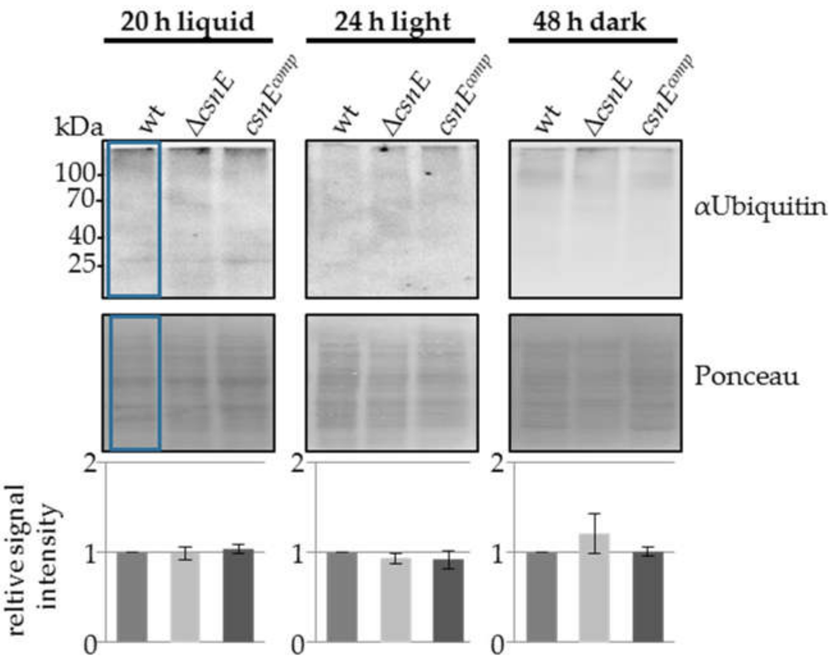

Figure S2. The amount of ubiquitinated proteins is not affected by CsnE. Fungal strains were grown for 20 h in liquid cultures and mycelia was harvested or afterwards shifted onto solid agar plates and incubated for 24 h in light to initiate asexual development or for 48 h in darkness to initiate sexual development at 37°C, respectively. Total cellular crude extracts were prepared from *A. nidulans* wild type, Δ*csnE* and *csnE*^{comp} strains to analyze the amount of ubiquitinated proteins. Proteins were separated on a 12 % SDS gel and afterwards blotted on a nitrocellulose membrane. Signals detected with the αUbiquitin antibody were normalized to Ponceau staining. The whole lane, framed in blue exemplarily for the wild type, was used for signal quantification. The standard error of the mean is shown of at least four biological replicates.

(a)

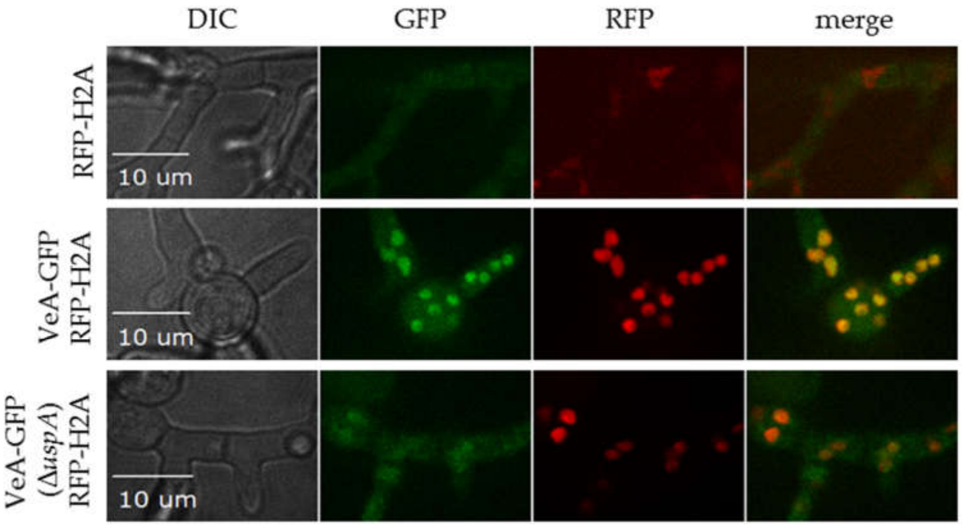

(b)

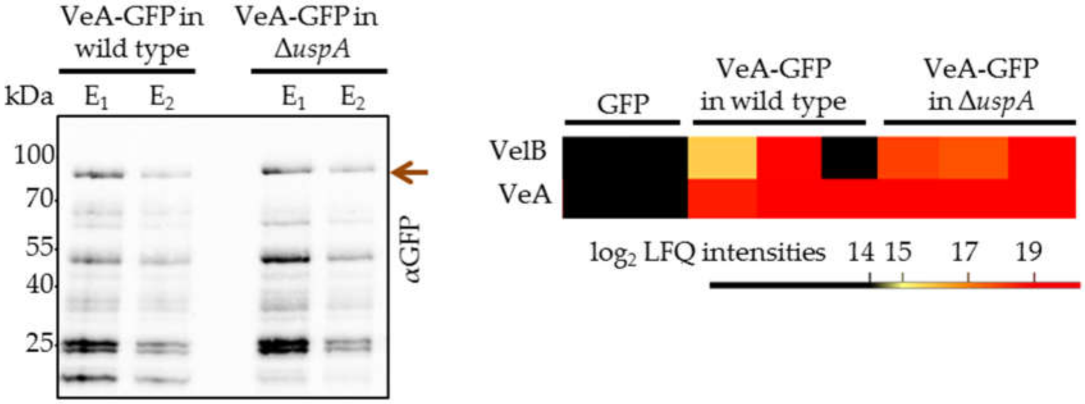

Figure S3. Localization of VeA and its interaction with VelB are independent of UspA. **(a)** VeA-GFP localization was analyzed with fluorescence microscopy of hyphae grown in liquid medium on cover slides for 20 h. VeA-GFP localizes in presence and absence of the deubiquitinase UspA inside nuclei that are visible in red due to RFP tagged histones. **(b)** GFP pull-down experiments with VeA-GFP as bait in wild type or Δ*uspA* background of cultures grown for 20 h in liquid medium were performed. Western blots revealed enrichment of VeA-GFP (indicated with red arrow) in the first two elution fractions (E1, E2). Co-purified proteins were identified with LC-MS and this revealed that the interaction of VeA with VelB is not dependent on UspA.

Table S1: Plasmids used in this study.
^{P}=promotor, ^{T}=terminator, ^{R}= resistance, ^{Af} = *Aspergillus fumigatus*, RM=recyclable marker cassette, *bleo* = *phleomycin*, *nat* = nourseothricin

Plasmid	Description	Reference
pUC19L	cloning vector, *amp*^{R}	Thermo Fisher Scientific
pBluescript KS	cloning vector, *amp*^{R}	Fermentas GmbH
pJET1.2	cloning vector, *amp*^{R}	Thermo Fisher Scientific
pME4313	BiFC vector containing *niaD*^{t}-*Swa*I-^{P}*niaD*/^{P}*niiA*-*Pme*I-*niiA*^{t}, *phleo*^{R}	[47]
pME4304	*six*-^{P}*xylP*:β-*rec*:*trpC*^{t}-*nat*^{R}-*six*	[47]
pME4601	^{P}*niaD:cYFP*, ^{P}*niiA:ANrcoA:neyfp*	[47]
pME4696	cloning vector containing *Pml*I restriction site-*natRM*-*Swa*I restriction site	this study
pME3857	^{P}*gpdA:mrfp:h2A:hisB*^{T}; *phleo*^{R}	[46]
pME3281	*phleo* cassette; shortened ^{P}*gpdA::ble::trpC*^{T}, shortened *phleo* cassette blunted into pBluescript, *bla*	[54]
pME4654	5'^{csnE}:*phleo*RM:3'^{csnE}	[28]
pME4701	5'^{csnE}- *csnE::nat*RM -3'^{csnE}	this study
pME4703	5'^{uspA}- *pyroA*^{Af} -3'^{uspA}	this study
pME4704	5'^{uspA}- *uspA* -3'^{uspA} in pME3281	this study
pME4706	5'^{uspA}- *uspA:gfp:*^{P}*gpdA:nat* -3'^{uspA}	this study
pME4707	5'^{uspA}- *uspA*^{AA}*:gfp:nat*RM -3'^{uspA}	this study
pME4708	^{P}*niaD*:*ceyfp*:*uspA*:*niaD*^{T} in *SwaI* site, ^{P}*niiA*:*neyfp*:*csnB*:*niiA*^{T} in *Pme*I site of pME4313	this study
pME4709	^{P}*niaD*:*ceyfp*:*niaD*^{T} in *SwaI* site, ^{P}*niiA*:*neyfp*:*csnB*:*niiA*^{T} in *Pme*I site of pME4313	this study
pME4710	^{P}*niaD*:*ceyfp*:*niaD*^{T} in *SwaI* site, ^{P}*niiA*:*neyfp*:*niiA*^{T} in *Pme*I site of pME4313	this study
pME4711	^{P}*niaD*:*ceyfp*:*uspA*:*niaD*^{T} in *SwaI* site, ^{P}*niiA*:*neyfp*:*csnF*:*niiA*^{T} in *Pme*I site of pME4313	this study
pME4712	^{P}*niaD*:*ceyfp*:*niaD*^{T} in *SwaI* site, ^{P}*niiA*:*neyfp*:*csnF*:*niiA*^{T} in *Pme*I site of pME4313	this study

pME4714	$5'^{veA}$- *veA:gfp:nat*RM-$3'^{veA}$	this study
pME4722	candA-N:candA-C:gfp, *natRM,* used as template for PCR amplification	[28]
pME4685	^{T}*niiA:yfp*^{N}:An_*candA-C1*^{P}*:niiA/niaD:NiiD*^{T}*:phleo*^{R} in pME3741, *bla,* used as template for PCR amplification	[28]
pME3741	BiFC vector, used as template	[54]
pME4715	*bla,* ^{P}*ADH:lexA:uspA*^{cDNA}*:ADH*^{T}*, HIS1, 2mm / two hybrid bait vector in Not*I restriction site	this study
pME2502	*csnA* cDNA of pME2987 *(Xho*I) in pJG4-5	[24]
pME2978	*csnB* cDNA of pME2988 *(Xho*I) in pJG4-5	[24]
pME2979	*csnC* cDNA of pME2989 *(Xho*I) in pJG4-5	[24]
pME2355	*csnD* cDNA of pME2990 *(Xho*I) in pJG4-5	[24]
pME2980	*csnE* cDNA of pME2991 *(Eco*RI) in pJG4-5	[24]
pME2981	*csnF* cDNA of pME2992 *(Eco*RI) in pJG4-5	[24]
pME2982	*csnG* cDNA of pME2993 *(Eco*RI) in pJG4-5	[24]
pME2983	*csnH* cDNA of pME2987 *(Eco*RI) in pJG4-5	[24]
pME2501	*csnA* cDNA of pME2987 *(Xho*I) in pEG202	[24]
pJG4-5 (pME3230)	*bla,* ^{P}*GAL1::B42::MCS::*^{T}*ADH, TRP1, 2mm / two-hybrid prey vector*	[55]
pEG202 (pME3229)	*bla,* ^{P}*ADH::lexA::MCS::*^{T}*ADH, HIS3, 2mm / two-hybrid bait vector*	[56]

Table S2. *A. nidulans* and *S. cerevisiae* strains used in this study. ^{P}=promotor, ^{T}=terminator, ^{R}=resistance Δ= deletion, $_{af}$ = *Aspergillus fumigatus, bleo = phleomycin, nat* = nourseothricin, :: = replacement of the gene locus, : = fusion

**Strain name**	**Genotype**	**Reference**
	A. nidulans strains	
AGB551	*ΔnkuA::argB, pyrG89, pyroA4, veA+*	[46]
AGB1014	*ΔnkuA::argB, pyrG89, pyroA4, veA+,*^{P}*gpdA:mrfp:h2A:hisB*^{T}*:nat*^{R}	[47]
AGB1066	*ΔnkuA::argB, pyrG89, pyroA4, veA+, veA::six*	[47]
AGB822	*ΔnkuA::argB, pyrG89, pyroA4, veA+, Δfbx23::pyrG*$_{Af,}$	this study
AGB1169	*ΔnkuA::argB, pyrG89, pyroA4, veA+, ΔcsnE::six*	this study
AGB1233	*ΔnkuA::argB, pyrG89, pyroA4, veA+, ΔcsnE::csnE:six*	this study
AGB1170	*ΔnkuA::argB, pyrG89, pyroA4, veA+,* ^{P}*niiA:cYFP:uspA:niiA*^{T}*,* ^{P}*niaD:nYFP:csnB:niaD*^{T}*, bleo*^{R}*,* ^{P}*gpdA:mrfp:h2A:hisB*^{T}*:nat*^{R}	this study
AGB1171	*ΔnkuA::argB, pyrG89, pyroA4, veA+,* ^{P}*niiA:cYFP:niiA*^{T}*,* ^{P}*niaD:nYFP:csnB:niaD*^{T}*, bleo*^{R}*,* ^{P}*gpdA:mrfp:h2A:hisB*^{T}*:nat*^{R}	this study
AGB1172	*ΔnkuA::argB, pyrG89, pyroA4, veA+,* ^{P}*niiA:cYFP:uspA:niiA*^{T}*,* ^{P}*niaD:nYFP:niaD*^{T}*, bleo*^{R}*,* ^{P}*gpdA:mrfp:h2A:hisB*^{T}*:nat*^{R}	this study
AGB1173	*ΔnkuA::argB, pyrG89, pyroA4, veA+,* ^{P}*niiA:cYFP:uspA:niiA*^{T}*,* ^{P}*niaD:nYFP:csnF:niaD*^{T}*, bleo*^{R}*,* ^{P}*gpdA:mrfp:h2A:hisB*^{T}*:nat*^{R}	this study
AGB1174	*ΔnkuA::argB, pyrG89, pyroA4, veA+,* ^{P}*niiA:cYFP:niiA*^{T}*,* ^{P}*niaD:nYFP:csnF:niaD*^{T}*, bleo*^{R}*,* ^{P}*gpdA:mrfp:h2A:hisB*^{T}*:nat*^{R}	this study
AGB1159	*ΔnkuA::argB, pyrG89, pyroA4, veA+, uspA:sgfp:*^{P}*gpdA:nat*^{R}	this study
AGB1161	*ΔnkuA::argB, pyrG89, pyroA4, veA+, uspA:sgfp:*^{P}*gpdA:nat*^{R}*,* ^{P}*gpdA:mrfp:h2A:hisB*^{T}*; phleo*^{R}	this study
AGB1162	*ΔnkuA::argB, pyrG89, pyroA4, veA+, uspA*^{AA}*:sgfp:six*	this study

AGB1163	*ΔnkuA::argB, pyrG89, pyroA4, veA+,* $uspA^{AA}$*:sgfp:six,* $^{P}gpdA$*:mrfp:h2A:*$hisB^{T}$; $phleo^{R}$	this study
AGB1156	*ΔnkuA::argB, pyrG89, pyroA4, veA+, ΔuspA::*$pyroA_{af}$	this study
AGB1157	*ΔnkuA::argB, pyrG89, pyroA4, veA+, ΔuspA::*$pyroA_{af}$*, uspA:*$bleo^{R}$	this study
AGB1164	*ΔnkuA::argB, pyrG89, pyroA4, veA+, ΔuspA::*$pyroA_{af}$*, veA:sgfp:six*	this study
AGB1165	*ΔnkuA::argB, pyrG89, pyroA4, veA+, veA:sgfp:six*	this study
AGB1166	*ΔnkuA::argB, pyrG89, pyroA4, veA+, ΔuspA::*$pyroA_{af}$*, veA:sgfp:six,* $^{P}gpdA$*:mrfp:h2A:*$hisB^{T}$; $phleo^{R}$	this study
AGB1167	*ΔnkuA::argB, pyrG89, pyroA4, veA+, veA:sgfp:six,* $^{P}gpdA$*:mrfp:h2A:*$hisB^{T}$:$bleo^{R}$	this study
AGB1234	*ΔnkuA::argB, pyrG89, pyroA4, veA+, Δfbx23::*$pyrG_{Af,}$ *veA:sgfp:six*	this study
***S. cerevisiae* strain**		
EGY48	*MAT trp1, his3, ura3, lexAops-LEU2*	[57]

Table S3: Oligonucleotides used for plasmid construction in this study.

Primer	Size	Sequence 5´→ 3
CM37	45mer	ATT CGA GCT CGG TAC GTT TAA ACC GAA ACG CTA TTT ATC CTG ATC
CM43	25mer	TTA GAT TTC TTT CAT TCT TTC TTT C
CM44	48mer	CCA AGC TTG CAT GCC GTT TAA ACT TTT TTT TGC TCC TTT TAT TTC TTT
CM48	35mer	GAA CAG AAC TTC CAG GTC AAG TCG TTC CTC TTC AC
CM94	18mer	CGT GGC GAT GGA GCG CAT
CM99	35mer	CCT ATA GGC CTG AGT TCA TGC GCT AAG TAG ACT CT
CM128	32mer	ACG CGG CCG CTC ATG TCG GGC TCA GAG AAC AA
CM129	31mer	CAG CGG GCC GCT CAG TCA AGT CGT TCC TCT T
CM138	46mer	CCA AGC TTG CAT GCC GTT TAA ACT AGA TAG AAT AAT ACG CAG ACA C
CM139	20mer	TCA TGT AGG CGG TAT TGC CC
CM140	21mer	ATA CCG CCT ACA TGA ACT CTG
CM141	20mer	TCT TTG GCG CGC GGA CAA TA
CM142	19mer	GCG CGC CAA AGA GCA TCG A
CM161	34mer	GCC ACG GGC GCG CCG TCG GGC TCA GAG AAC AAG C
CM162	23mer	TCA CAT GAT ATA GAC GTT GTG GC
CM163	37mer	CGC TCC ATC GCC ACG TCA GAC GAC GAT GAT TTC ATG C
CM164	20 mer	TTA GAA CAG GCC CGT CTT CA
CM165	20 mer	TCA CTT GTA CAG CTC GTC CA
CM166	35 mer	CGC TCC ATC GCC ACG CCA GAC GAA GCC ATA TCC AT
CM167	19mer	TTA TCC GAG TGC CAC GCC A
KT142	39mer	ATA ATA TGG CCA TCT AAG AAT TCT GCC GGC GTT TAT TTG
KT197	39mer	ATC GAT AAG CTT GAT GTT TAA ACT GGA GTG CCT TTC GTC
KT198	39mer	CTG CAG GAA TTC GAT GTT TAA ACA TTC TGG CTC GTC TGC
SR18	33mer	GGT GGT AGC GGT GGT GTG AGC AAG GGC GAG GAG
SR20	38mer	CTA TAG GCC TGA GTG CTA CTT GTA CAG TTC GTC CAT GC
SR44	35mer	ACC ACC GCT ACC ACC ACG CAT GGT GGC AGG CTT TG
AMK82	20mer	CTG GAA GTT CTG TTC CAG GG

AMK85	45mer	CCA AGC TTG CAT GCC ATT TAA ATC TAC TTG TAC AGT TCG TCC ATG
AMK86	37mer	GAA CTG TAC AAG TAG ATT TGG CGG CTC TGA GGT GCA G
AMK163	18mer	ATG GTG AGC AAG GGC GAG
AMK168b	22mer	ATG GCC GAC AAG CAG AAG AAC G
AMK169b	45mer	CGG GCG GCC CGT GGC GAT GGA GCG CTT GTA CAG CTC GTC CAT GC
AL39	43mer	ATT CGA GCT CGG TAC GTT TAA ACA CTT ACT CGT CCA CAA GCT T
AL40	38mer	ACC TAT AGG CCT GAG TGA TGA TTG TCA GGT GGG GAT AT
AL47	36mer	AGT TGA GCA TAA TAT CAG ATG ATG AGA CGA TCT ATG
AL48	41mer	CCA AGC TTG CAT GCC GTT TAA ACT GGG GAC GAT ATG ATC AG
SI27	32mer	CAG GTC ACC TGG TAT CAT GGT TGT TGG GTC TC
SI28	30mer	TAC ATA TGA GCA TCC ACA TGA TCG ACA GCC
SI29	32mer	CAG GTC ACC ATT ACC CCA GCA TTG ATC AGA CC
SI30	30mer	CAG GTC ACC ATC GGC CGC GGA ATC GCT AAC
SI31	30mer	CAC ATA TGT ACA TGG TTT GGG GCA GAG TGT
SI32	34mer	CAC ATA TGT AGA TAG AAT AAT ACG CAG ACA CAG G
EB2	27mer	CTA CTT GTA CAG TTC GTC CAT GCC GTG
flip-1	43mer	ACC TAT AGG CCT GAG ATT TAA ATA TCG AAT TCC TGC AGC CCG G
flip-2	43mer	ATA ATA TGG CCA TCT CAC GTG ATC AAG CTT ATC GAT ACC GTC G

Table S4: Oligonucleotides used for qRT experiments.

Primer	Size	Sequence 5´--> 3
CM_RT_1	22mer	CGA GGC TGA GCA GGA TGT AGA A
CM_RT_2	22mer	TGG TGT TGT TCT GGG TTC CTG T
CM_RT_45	22mer	CCA AGA TTC CCC TCA ACA CAT C
CM_RT_46	22mer	CAT CGG AGC CAT TAG GAC TTT G
CM_RT_47	22mer	ACA GTT TCC ACA GCG ACT TTC C
CM_RT_48	22mer	ATG TCT CGG AAA CGG GGT AGT T
CM_RT_49	22mer	CTC CTA CCC TCA ATC GTG ATG C
CM_RT_50	22mer	TGT TTC TTG AGC TCG TCA GTG C
CM_RT_51	22mer	CAC ATT GTC CAA GCA CCC TGT A
CM_RT_52	22mer	ACA GAT TCG AAG GAG CCA TCA G
CM_RT_53	22mer	GAC GTA CAG GTT CAG GCG AAG A
CM_RT_54	22mer	GTT TCT TGA TCC GGA GCT GCT T
CM_RT_55	22mer	GAA GGA GCA GGA GCA GGA GAA C
CM_RT_56	22mer	AGG AGC GGG AGA GGG TAG ACT T
JG680	22mer	GAC TGG ATT GAG ACG GAG CAA A
JG681	22mer	TTC AGG ACA AGG AAG ACG GAT G
JG682	21mer	CCG AGA CAG ATG CGG ACA GAT
JG683	22mer	CAA CAG GCA CCC AAT CCA CTA A
JG684	22mer	ACC CGC ACA CCT GGA ACA TAA C
JG685	23mer	GAA TAC ACA TCA CGC TCC CAA CA
JG686	22mer	GTT TCT TCG GCG GTG CTC TAA T
JG687	22mer	CAG TTG GAA TGG TGG GAA TGA G
JG688	22mer	CCA GCG GAG AAG AGG CAG ATT A
JG689	22mer	CAT AGA CGA AGC GAA AGG TGG A
JG690	22mer	TCA CCT ACA AGG ACC CCA ACA C
JG691	21mer	CCC GAA TGA CGC AAA AGA AAG
JG692	22mer	GAG TCC CTC GCC GTA TCA ACT C
JG693	22mer	CCT ATG ATC GCT TGT GGG GTC T

JG1445	22mer	TCT CTC GCC TTA CAG TGA ATG A
JG1446	22mer	AGT GAT GGA GTG CTG AGG TTC T
JG1478	21mer	TTG TTG ACG GGA CGA CTG TAG
JG1479	22mer	TTT GTG CGT GTA GTG AGG GTA G
KT312	20mer	TCT CGA GCT TGC TGG AAA CG
KT313	20mer	CAC CCT GGG CAA TAG TGA CG
JS_RT_203	19mer	GCC AAG CCT AAC GAG AAG C
JS_RT_204	20mer	GGG AAT GAA ACG GGA AGA GT

Table S5: Functional groups of protein identified in GFP pull-downs of UspA-GFP and UspA^{AA}-GFP. GFP pull-downs of UspA-GFP and UspA^{AA}-GFP of cultures that were grown vegetatively in light at 37°C. Elution fractions were digested with trypsin and peptides were identified in LC/MS-MS analyses. Identified proteins were present in at least two out of three biological replicates with at least two unique peptides and log2 LFQ intensity of 20. Description of proteins derive from information on UniProt, AspGD or FungiDB [56,57]. Domain predictions for uncharacterized proteins were performed with NCBI CD domain prediction tool [59].

Systematic Name	Gene Name	Description	UspA-GFP	UspA^{AA}-GFP
**Primary metabolism**				
AN3524	-	NAD binding Rossmann fold oxidoreductase	X	X
AN7199	-	galactonate metabolism	X	X
AN0723	-	domains of sulfotransfer superfamily	X	X
AN7590	-	mannitol dehydrogenase	X	X
AN1318	-	tyrosinase, catechol oxidase activity	X	X
AN6521	*lysF*	homoaconitase, mitochondrial	X	X
AN4956	*AHAS-L*	acetolactate synthase	X	X
AN8782	-	esterase, S-formylglutathione hydrolase	X	X
AN6952	-	S-adenosyl dependent methyltransferase	X	X
AN7895	*cipB*	zinc-binding alcohol dehydrogenase domain	X	X
AN1023	*sagA*, *end3*	actin cytoskeleton-regulatory complex protein,	X	X
AN7111	*foxA*	multifunctional beta-oxidation protein	X	X
AN5311	-	tyrosinase, catechol oxidase activity	X	X
AN7334	-	predicted role in metabolic processes	X	X
AN3331	-	phosphohydrolase superfamily		X
AN5328	-	GPI anchored dioxygenase		X
AN3616	-	monooxygenase		X

Systematic Name	Gene Name	Description	UspA-GFP	UspA^{AA}-GFP
		**Primary metabolism**		
AN1689	-	aldehyde dehydrogenase family		X
AN1882, AN6753	-	NADH-dependent flavin oxidoreductase		X
AN1602	*eglD*	beta-1,4-endoglucanase		X
AN2947	-	1-phosphatidylinositol-4,5-bisphosphate phosphodiesterase	X	
AN5883	*metF*	Methylenetetrahydrofolate reductase	X	
AN2493	-	gluconate metabolism	X	
		**Nuclear transport**		
AN0906	*kapB*	nuclear transport protein	X	X
AN3877	-	domains of NTF2 like superfamily	X	X
AN6734	kapF	karyopherin, nuclear receptor	X	X
AN5376	-	domains of NTF2 like superfamily	X	X
AN6978	*rcc1*	chromatin associated guanine nucleotide exchange factor	X	X
		**Development**		
AN10311	*mnpA*	hyphal cell wall mannoprotein	X	X
AN5635	*treB*	neutral trehalase	X	X
AN2523	*chsB*	chitin synthase B	X	X
AN6709	*hypB*	guanyl-nucleotide exchange factor, hyphal morphogenesis	X	X
AN8333	*phiA*	phialide development		X
		**Transcriptional processing**		
AN5452	-	pre-mRNA-splicing factor	X	X
AN1205	-	prefoldin subunit 5, regulation of transcriptional elongation	X	X

Systematic Name	Gene Name	Description	UspA-GFP	UspA^{AA}-GFP
		**Transcriptional processing**		
AN5894	-	Pol II transcription elongation factor subunit Cdc73	X	X
AN7680	-	domains of SMC superfamily, structural maintenance of chromosomes		X
AN0646	-	DNA/RNA helicase activity		X
AN2007	-	mRNA binding, splicing		X
AN4965	-	putative Ccr4-Not transcription complex subunit		X
AN3955	-	RNA metabolic process	X	
AN11128	-	putative RNA polymerase II transcription elongation factor	X	
AN7480	-	Differentiation regulator, Nrd1, RNA binding	X	
AN4024	-	RNA maintenance of telomere capping protein 1	X	
		**Ubiquitin-proteasome system**		
**AN6354**	**characterized in this study**	**Ubiquitin carboxyl-terminal hydrolase, UspA**	X	X
AN7422	-	Ubiquitin carboxyl-terminal hydrolase, UspF	X	X
AN2000	*ubi4*	polyubiquitin		X
		**Zomes**		
AN7540	-	Eukaryotic translation initiation factor 3 subunit D	X	X
AN10519	-	Proteasome regulatory particle subunit	X	X
		**Signaling**		
AN1545	*pabA*	Protein phosphatase PP2A regulatory subunit B	X	X
AN12477	-	GTP binding, GTPase activity	X	X

Systematic Name	Gene Name	Description	UspA-GFP	$UspA^{AA}$-GFP
**Signaling**				
AN10691	-	GTP binding domains	X	X
AN1867	*phoB*	Serine/threonine kinase		X
**Uncharacterized proteins**				
AN0860	-		X	X
AN3121	-	conserved glutamic acid-rich protein	X	X
AN10518	-		X	X
AN2647	-		X	X
AN3709	-	CRAL/TRIO domain protein	X	X
An3673	-			X
AN4650	-	conserved serine-proline rich region		X

Additional References:

93. Harper, S.; Besong T.M.; Emsley J.; Scott, D.J.; Dreveny I. Structure of the USP15 N-terminal domains: a β-hairpin mediates close association between the DUSP and UBL domains. *Biochemistry*. **2011**, *50*, 7995-8004, doi: 10.1021/bi200726e
